# Geographic footprints of life expectancy inequalities in the state of Geneva, Switzerland

**DOI:** 10.1038/s41598-021-02733-x

**Published:** 2021-12-02

**Authors:** Anaïs Ladoy, Juan R. Vallarta-Robledo, David De Ridder, José Luis Sandoval, Silvia Stringhini, Henrique Da Costa, Idris Guessous, Stéphane Joost

**Affiliations:** 1 Laboratory of Geographic Information Systems (LASIG), School of Architecture, Civil and Environmental Engineering (ENAC), Ecole Polytechnique Fédérale de Lausanne (EPFL), Lausanne, Switzerland; 2 Group of Geographic Information Research and Analysis in Population Health (GIRAPH), Geneva, Switzerland; 3 Faculty of Medicine, University of Geneva, Geneva, Switzerland; 4 Unit of Population Epidemiology, Department of Primary Care, Geneva University Hospitals, Geneva, Switzerland; 5 Department of Oncology, Geneva University Hospitals, Geneva, Switzerland; 6 Division of Primary Care Medicine, Department of Primary Care, Geneva University Hospitals, Geneva, Switzerland; 7 University Centre for General Medicine and Public Health, University of Lausanne, Lausanne, Switzerland; 8 Réseau Delta, HMO, Geneva, Switzerland; 9 La Source School of Nursing, University of Applied Sciences and Arts Western Switzerland (HES-SO), Lausanne, Switzerland

**Keywords:** Public health, Epidemiology

## Abstract

Though Switzerland has one of the highest life expectancies in the world, this global indicator may mask significant disparities at a local level. The present study used a spatial cluster detection approach based on individual death records to investigate the geographical footprint of life expectancy inequalities in the state of Geneva, Switzerland. Individual-level mortality data (n = 22,751) were obtained from Geneva’s official death notices (2009–2016). We measured life expectancy inequalities using the years of potential life lost or gained (YPLLG) metric, defined as the difference between an individual’s age at death and their life expectancy at birth. We assessed the spatial dependence of YPLLG across the state of Geneva using spatial autocorrelation statistics (Local Moran’s I). To ensure the robustness of the patterns discovered, we ran the analyses for ten random subsets of 10,000 individuals taken from the 22,751 deceased. We also repeated the spatial analysis for YPLLG before and after controlling for individual-level and neighborhood-level covariates. The results showed that YPLLG was not randomly distributed across the state of Geneva. The ten random subsets revealed no significant difference with the geographic footprint of YPLLG and the population characteristics within Local Moran cluster types, suggesting robustness for the observed spatial structure. The proportion of women, the proportion of Swiss, the neighborhood median income, and the neighborhood median age were all significantly lower for populations in low YPLLG clusters when compared to populations in high YPLLG clusters. After controlling for individual-level and neighborhood-level covariates, we observed a reduction of 43% and 39% in the size of low and high YPLLG clusters, respectively. To our knowledge, this is the first study in Switzerland using spatial cluster detection methods to investigate inequalities in life expectancy at a local scale and based on individual data. We identified clear geographic footprints of YPLLG, which may support further investigations and guide future public health interventions at the local level.

## Introduction

Health inequalities, defined as differences in the population’s health status^1^, remain a major challenge in public health^2,3^. Individuals more socioeconomically deprived usually face poorer health conditions and are at higher risk of presenting a premature death^4–7^. Premature death, evaluated through Years of Potential Life Lost (YPLL), which estimates the years a person did not live compared to an arbitrary age, is one of the most widely used mortality-based indicators to measure the population’s health^8–10^. This indicator was first introduced by Mary Dempsey^11^ to contrast the results obtained with mortality rate measurements in tuberculosis control.

Compared to other countries, Switzerland’s health inequalities for all-cause mortality are below average^9,12^. Nevertheless, these statistics constitute a global estimation of the health situation at the country level, and substantial regional differences of mortality and socioeconomic status have been reported in Switzerland at the neighborhood level^13,14^.

Evidence shows that neighborhood conditions (i.e., social, economic, and physical) have an influence on the health of individuals, independent of personal characteristics^15–17^. Spatial analyses are therefore valuable for revealing geographic patterns in health inequalities, identifying populations at risk, and guiding public health interventions at local scales^18,19^. In the last decade, increasing evidence suggests strong patterns in mortality indicators across geographical space, both at large^20–22^ and small geographic scales^23–26^. In addition, the spatial structure of premature mortality has been found to be significantly associated with deprivation status^20–26^, immigrant population size^23^, and multiple environmental features (e.g., pollution, greenspace, walkability)^24–26^.

These past analyses have been performed at aggregated levels, such as at county- or neighborhood-levels, making these results sensitive to variations in scale, which is commonly referred to as the modifiable areal unit problem (MAUP)^27^.

Furthermore, studies assessing the spatial distribution of mortality inequalities tend to focus only on premature mortality. Unfortunately, this indicator may smooth out mortality inequalities as it considers only individuals in the population that die before a certain age and ignores individuals living longer than expected.

We therefore sought to investigate mortality inequalities at a fine scale in the state of Geneva, using death records for 22,751 individuals from 2009 to 2016, with data georeferenced at the residential address. The outcome variable used here is an adaptation of the YPLL indicator, “years of potential life lost or gained” (YPLLG), and is defined as the difference (positive or negative) between age at death and life expectancy at birth (LEB). We believe that this indicator better captures mortality inequalities across geographical space. We assessed the spatial structure of YPLLG using spatial autocorrelation statistics, following which we also investigated the influence of individual-level and neighborhood-level covariates on the geographic footprint of YPLLG.

## Methods and materials

### Death notice data

Raw data included death records from the state of Geneva of 49,628 individuals between 1908 and 2017, with personal information of the deceased including name, date of birth, civil status, nationality (Swiss/non-Swiss), date of death, and residential address. Data were collected through web scraping of the official and publicly available death notices published in the *Feuille d’avis officielle (FAO)* until 2017 (Republic and Canton of Geneva, https://fao.ge.ch). Their use did not imply a request for authorization from the ethics committee of the canton of Geneva as the research protocol does not study disease, neither the structure nor the functioning of the human body (Federal Act on Research involving Human Beings, article 2). Moreover, the research protocol respects the Swiss Federal Act on Data Protection (Art. 22) specifying specifically that personal data can be processed for purposes not related to specific persons, and in particular for research, planning and statistics if the data is rendered anonymous as soon as the purpose of the processing permits, and if the results are published in such a manner that the data subjects may not be identified. For our analyses we only retained deaths from 2009 to 2016 (n = 27,889; 56.2% of raw data), years where we have information consistent with the number of deaths reported by the Cantonal Statistical Office, OCSTAT (www.ge.ch/statistique). From this subset, we further removed duplicated entries, individuals with missing date of birth, date of death, or nationality (n = 408; 1.5%), as well as individuals living outside the state of Geneva (n = 2094; 7.5%), and individuals that could not be georeferenced (n = 1025; 3.7%).

Due to the lack of specific gender information in the database, we used the genderize.io API for name-to-gender inference as it shows a correct performance rate compared to other web services^28^. This approach is commonly used in gender inequality research, such as investigating women’s representation in academic literature^29,30^. The API returns the gender most commonly associated with a given first name, along with confidence parameters. With this process, we recovered gender for 94.2% of individuals in our dataset (1611 observations were removed).

After data filtering, we were left with 22,751 individuals (81.6% of 2009–2016 deceased) for further analysis.

The study was carried out in accordance with the relevant guidelines and regulation.

### Mortality indicators

*(Cohort) life expectancy at birth (LEB)* represents the average lifespan of a group of individuals born in any given year, considering the observed and forecasted evolution of death rates through their lifetime^31^. Official estimates of cohort life expectancy were extracted from the 1900–2030 Swiss cohort life tables (FSO, https://www.bfs.admin.ch/), calculated from a model developed by Jacques Menthonnex^32^. Cohort LEB was preferred over the traditional period LEB as we believe that it better captures changes in mortality conditions across a lifetime (see Supplementary Fig. S4). LEB was attributed to the deceased based on year of birth and gender.

*Years of potential life lost (YPLL)* estimates the years a person did not live compared to an arbitrary age (usually 75)^8–10^ or compared to the individual’s LEB^11^. Note that this indicator does not consider the years a person may live beyond this age cut-off.

*Years of potential life lost or gained (YPLLG)* is defined as the difference in years between the age at death and the individual’s LEB. Positive values of YPLLG capture the years of life ‘gained’, while negative YPLLG values reflect the potential years of life ‘lost’, as defined by Dempsey^11^.

### Neighborhood-level characteristics

To assess the influence of neighborhood characteristics on the spatial distribution of YPLLG, we included yearly data of neighborhood socioeconomic status and median population age for the period between 2009 and 2016. Both indicators were available at the statistical subsector level (n = 475), a geographic unit smaller than that of municipality, which is used by the state of Geneva for the diffusion of local aggregated statistical data^33^.

The neighborhood socioeconomic status was measured using the median annual neighborhood household income, which was obtained via a request from the Cantonal Statistical Office, OCSTAT (C. Stohr, personal communication, 2020). The transmitted data excluded unmarried individuals (i.e., single, divorced, widowed) from the calculation of the annual neighborhood income. The reasons given by the Statistical office were that: (1) their taxable income is not a good indicator of their quality of life, and (2) the information of non-taxable income such as social assistance, which constitutes a significant part of unmarried taxpayers’ income, was not available before 2014.

The neighborhood population median age was estimated from the resident population by 5-year age groups, with a final open class of 100+. This information was obtained from the Cantonal Statistical Office website (https://www.ge.ch/statistique/domaines/01/01_01/tableaux.asp#4). As the calculation of median from grouped data requires classes with equal sizes, we assumed that individuals were not living longer than 105 years.

We assigned neighborhood-level characteristics to each individual based on the registered residential address at the date of the death. If data were not available for a specific neighborhood, we used the nearest neighborhood value.

### Statistical analysis

We investigated the spatial structure of YPLLG across the state of Geneva using the Local Moran statistic^34^. The statistic relies on a measure of spatial dependence (or spatial autocorrelation), i.e., how similar observations tend to be within a specific neighborhood (spatial lag), and identifies local clusters of low and high YPLLG values. By differentiating the relationships between individuals and their surroundings into five categories, the Local Moran approach allows for precise interpretation of the spatial structure of a given phenomenon.

We decided to analyze the YPLLG variable within a 1200-m buffer (spatial lag) around each individual’s residential address. This methodological choice was supported by similar epidemiological studies conducted in the state of Geneva^35,36^.

For each residential address, the correlation between the observed variable and the mean of this variable in a given neighborhood (spatial lag) was calculated. The standardized scatterplot of this relationship allows to identify four distinct types of spatial association: (1) High–High clusters (dark green dots in the maps) represent individuals with high YPLLG values (i.e., that live longer than expected) surrounded by individuals with high YPLLG values; (2) Low–Low clusters (dark purple dots in the maps) represent individuals with low YPLLG values (i.e., that live shorter than expected) surrounded by individuals with low YPLLG values, (3) Low–High spatial outliers (light purple dots in the maps) represent individuals with high YPLLG surrounded by individuals with low YPLLG, and (4) High–Low spatial outliers (light green dots in the maps) represent individuals with low YPLLG surrounded by individuals with high YPLLG.

To assess whether or not the null hypothesis of no spatial association can be rejected, we performed a significance test using 99,999 Monte-Carlo permutations where the value *y*_*i*_ at a specific location *i* is held fixed for each step and the location of its neighboring values are randomly permuted^34^. Pseudo p-values were then calculated as the probability of obtaining a local Moran’s I larger than observed^37^. To consider the effects of simultaneous multiple comparisons^38^, we applied a Bonferroni correction for an overall alpha level of 0.1, resulting in an individual significance level of 1e-5. Non-significant locations (i.e., with pseudo p-value > 1e−5) are shown in white on the maps.

To evaluate the degree to which neighborhood-level characteristics, such as socioeconomic status or population age, explain the spatial structure of YPLLG, we performed the same analysis on adjusted YPLLG values obtained with a median regression. This regression model is preferred to the traditional Ordinary Least Square model when the outcome variable does not follow a normal distribution^39^, which is the case of YPLLG (Supplementary Fig. S1). We also included the nationality (Swiss/Non-Swiss) in the regression model to control for potential confounders. As gender and age were used to calculate an individual’s LEB, they were not included as dependent variables. More detailed information about the regression model is provided in the Supplementary Materials (Eq. S1).

Methodological and computational issues may arise from conducting spatial statistics on such large datasets. First, the Bonferroni bound (defined as α/n, where α = 0.1, and n is the number of observations) requires 9e+5 permutations to be applied to the dataset. Second, as we are using point data, we cannot guarantee that the spatial structure of mortality discovered with Local Moran’s statistic are not entirely due to the configuration of these specific data points for the period between 2009 and 2016. Therefore, we replicated the analysis (both for the raw and adjusted YPLLG models) on ten random subsets, each containing 10,000 observations drawn from the 22,751 deceased. With this method, we could perform enough permutations to apply a Bonferroni correction while ensuring the robustness of the discovered spatial structure. Description of samples, characteristics of the spatial weights, and regression results for the ten subsets can be found in Supplementary Tables S1–S3. Since the spatial structure of YPLLG was similar across subsets, only the maps of subset 8 are shown in the paper for descriptive purposes. However, the results for the other subsets can be found in the Supplementary Figs. S2 and S3.

For both the raw and adjusted YPLLG models, we summarized the results of the ten random subsets by calculating the range, mean, and standard deviation of population characteristics within each cluster type (i.e., Not significant, High–High, Low–Low, High–Low, Low–High). These population characteristics include the number of individuals within each cluster type, gender, nationality, neighborhood household income and population age, YPLLG value, and individual’s YPLL. The YPLL was calculated using a 75-year cut-off^40,41^. We used Tukey’s HSD test to compare all the possible pairs of means between each of the Local Moran cluster types to identify significant differences in population characteristics.

Spatial analyses were performed in R using the rgeoda package^42^.

### Ethics approval, consent to participate and consent for publication

Data used for analysis were publicly available through the *Feuille d’avis officielle* (FAO) until July 2017 (Republic and Canton of Geneva, https://fao.ge.ch). The use of these data did not imply a request for authorization from the ethics committee of the canton of Geneva. Indeed, the research protocol used does not study disease, neither the structure nor the functioning of the human body (Federal Act on Research involving Human Beings, article 2). Moreover, the research protocol respects the Swiss Federal Act on Data Protection (Art. 22) specifying that personal data can be processed for purposes not related to specific persons, and in particular for research, planning and statistics, if: (a) the data is rendered anonymous, as soon as the purpose of the processing permits; (b) the recipient only communicates the data to third parties with the consent of the body that transmitted them, and (c) the results are published in such a manner that the data subjects may not be identified.

## Results

### Profile of the deceased

Of the 22,751 deceased included in our analysis, 12,125 (53.3%) were women, and 18,101 (79.6%) were Swiss. Individuals lived in neighborhoods with a median household income of 128,012 ± 41,014 CHF per year and a median population age of 42.96 ± 9.70. The YPLLG distribution among individuals was negatively skewed, with a mean value of 5.19 ± 20.12 (min: − 93.6, max: 49.9, median: 9.3) (Supplementary Fig. S1). The mean lifespan for the dataset was 79.42 ± 15.34 years.

Median YPLLG was significantly lower for men (8.2) than for women (9.9, p < 0.001), and lower for non-Swiss (3.4) than for Swiss (10.6, p < 0.001). Men tended to live in younger neighborhoods (i.e., based on the median age of the neighborhood population) than women (men: 40.4 vs. women: 40.8 years, p < 0.001). Similar was found for non-Swiss compared with Swiss (non-Swiss: 40.0 vs. Swiss: 40.8 years, p < 0.001). Non-Swiss were also located in more deprived neighborhoods than Swiss (non-Swiss: 111,076 vs. Swiss: 115,733 CHF per year, p < 0.001), No significant differences were found in neighborhood income between the genders (men: 114,208 vs. women: 115,226 CHF per year, p = 0.55).

### Characteristics of individuals within clusters

To assess whether differences existed between individuals within and outside the clusters discovered by the Local Moran analysis, we compared summary statistics for individuals’ gender, nationality, median neighborhood income and age, YPLLG, and YPLL taken from the ten random replications.

For the raw YPLLG model (Table 1), the Local Moran analysis detected a mean of 3502 (35%) individuals showing spatial dependence (i.e., that have a pseudo p-value < 1e−5) for the ten random subsets, including 1,445 individuals belonging to high YPLLG clusters, 779 to low YPLLG clusters, 525 to Low–High spatial outliers, and 751 to High–Low spatial outliers. On average, individuals in high YPLLG clusters lived 19.21 years longer than expected (i.e., according to their Life Expectancy at Birth), individuals in low YPLLG clusters lived 15.06 years less than expected, and individuals showing no spatial dependence lived 4.40 years longer than expected. We observed similar trends when comparing the mean YPLL value between High–High (mean YPLL: 0) and Low–Low clusters (mean YPLL: 11.70). Other population characteristics significantly differed between clusters of low or high YPLLG values and locations showing no spatial dependence. In particular, the median annual neighborhood household income was significantly higher for individuals in high YPLLG clusters (122,870 CHF) than those in low YPLLG clusters (111,146 CHF, p < 0.001), as well as the neighborhood population median age (High–High: 54.0, Low–Low: 38.9, p < 0.001), the prevalence of women (High–High: 61.5%, Low–Low: 45.7%, p < 0.001), and the prevalence of Swiss (High–High: 86.0%, Low–Low: 70.4%, p < 0.001). We observed no significant differences in population characteristics between the ten random subsets.

**Table 1:** Characteristics of individuals within each Local Moran cluster type for the raw YPLLG model.

		Not significant	High–High	Low–Low	Low–High	High–Low
N		5981–7018	1161–1715	627–883	385–615	599–835
		6497.9 (65.0%)	1445.4 (14.5%)	778.5 (7.8%)	524.9 (5.2%)	751.1 (7.5%)
		± 270.24	± 143.16	± 86.84	± 74.84	± 75.73
Mean YPLLG		3.94–4.86	18.74–19.51	− 15.91 to − 14.22	− 11.77 to − 9.90	16.54–17.30
		4.40	19.21	− 15.06	− 10.56	16.95
		± 0.28	± 0.29	± 0.51	± 0.66	± 0.26
Gender	Women	3084–3664	722–1059	295–418	224–355	304–466
		3374.0 (51.9%)	889.3 (61.5%)	355.1 (45.7%)	298.9 (57.1%)	398.0 (52.9%)
		± 159.29	± 90.48	± 39.10	± 39.91	± 47.27
	Men	2897–3354	439–656	332–491	161–275	295–390
		3123.9 (48.1%)	556.1 (38.5%)	423.4 (54.3%)	226.0 (42.9%)	353.1 (47.1%)
		± 121.71	± 56.51	± 52.58	± 38.86	± 31.46
Nationality	Non swiss	1260–1422	174–239	184–269	94–149	124–156
		1343.5 (20.7%)	202.2 (14.0%)	230.5 (29.6%)	126.1 (24.1%)	139.2 (18.6%)
		± 48.01	± 18.78	± 30.28	± 17.51	± 10.06
	Swiss	4721–5619	987–1476	443–632	291–476	475–702
		5154.4 (79.3%)	1243.2 (86.0%)	548.0 (70.4%)	398.8 (75.9%)	611.9 (81.4%)
		± 229.96	± 125.47	± 58.42	± 59.53	± 68.64
Mean YPLL		4.27–4.64	0.00–0.00	11.01–12.32	7.60–9.22	0.00–0.00
		4.43	0.00	11.70	8.18	0.00
		± 0.13	± 0.00	± 0.38	± 0.56	± 0.00
Mean neighborhood household income		131,050–133,722	118,996–126,142	107,923–113,317	128,348–137,893	112,682–119,932
		132,267.2	122,870.5	111,145.6	133,403.8	116,913.6
		± 833.92	± 2677.39	± 1851.32	± 3121.58	± 2445.51
Mean neighborhood population age		40.81–41.42	52.97–55.70	38.70–39.01	45.72–48.06	39.03–39.43
		41.17	53.99	38.87	46.56	39.16
		± 0.22	± 0.78	± 0.11	± 0.75	± 0.12

For each variable, the table shows range, mean, and standard deviation, calculated across the ten subsets. The totals per category may not be equal to 10,000 due to neighborless individuals who were excluded from the Local Moran’s analysis.

Table 2 shows the summary results for the adjusted YPLLG model, in which we explicitly accounted for the effect of individual-level and neighborhood-level covariates with a median regression model. We observed fewer significant locations (1993; 20%) compared to the raw YPLLG model, but we found similar trends in population characteristics within each cluster type. For the ten random subsets, the analysis detected, on average, 752 individuals belonging to high YPLLG clusters, 375 to low YPLLG clusters, 406 to Low–High spatial outliers, and 457 to High–Low spatial outliers. Individuals in high YPLLG clusters lived, on average, 9.09 years longer than expected, while individuals in low YPLLG clusters lived 23.38 years shorter than expected, and individuals showing no spatial dependence lived 3.76 years shorter than expected. Median annual neighborhood household income was significantly lower for individuals in low YPLLG clusters (111,818 CHF) than in high YPLLG clusters (131,410 CHF, p < 0.001). Spatial clusters consisting of individuals living shorter than expected (i.e., low YPLLG clusters) were also found in younger neighborhoods (Low–Low: 38.97, High–High: 46.68, p < 0.001). Significant differences between low and high clusters were also observed for gender (58.7% of women in High–High clusters, and 46.5% of women in Low–Low clusters, p < 0.001), and nationality (83.6% of Swiss in High–High clusters, and 76.1% of Swiss in Low–Low clusters, p < 0.001).

**Table 2:** Characteristics of individuals within each Local Moran cluster type for the adjusted YPLLG model.

		Not significant	High–High	Low–Low	Low–High	High–Low
N		7271–8866	466–1097	179–500	224–550	263–579
		8007.1 (80.1%)	752.3 (7.5%)	375.3 (3.8%)	405.8 (4.1%)	457.3 (4.6%)
		± 431.16	± 172.92	± 101.42	± 94.11	± 106.13
Mean adjusted YPLLG		− 4.10 to − 3.47	8.46–9.74	− 24.32 to − 21.70	− 20.84 to − 18.71	8.58–9.55
		− 3.76	9.09	− 23.38	− 19.75	9.03
		± 0.22	± 0.39	± 0.90	± 0.76	± 0.36
Gender	Women	3824–4692	279–664	88–237	134–301	140–301
		4226.9 (52.8%)	443.3 (58.7%)	173.5 (46.5%)	229.3 (56.9%)	242.3 (53.1%)
		± 232.73	± 113.66	± 46.01	± 49.00	± 54.87
	Men	3447–4174	187–433	91–263	90–249	123–291
		3780.2 (47.2%)	309.0 (41.3%)	201.8 (53.5%)	176.5 (43.1%)	215.0 (46.9%)
		± 209.30	± 62.83	± 57.28	± 47.78	± 52.78
Nationality	Non swiss	1528–1788	80–169	41–121	42–98	60–143
		1645.5 (20.6%)	122.0 (16.4%)	90.3 (23.9%)	73.6 (18.1%)	110.1 (24.1%)
		± 79.34	± 23.84	± 26.72	± 18.30	± 26.39
	Swiss	5743–7078	386–928	138–389	182–452	203–440
		6361.6 (79.4%)	630.3 (83.6%)	285.0 (76.1%)	332.2 (81.9%)	347.2 (75.9%)
		± 355.62	± 150.50	± 76.78	± 76.84	± 81.00
Mean YPLL		4.02–4.41	0.00–0.02	11.56–13.40	7.83–9.72	0.02–0.05
		4.23	0.01	12.59	8.65	0.04
		± 0.13	± 0.00	± 0.61	± 0.62	± 0.01
Mean neighborhood household income		127,443–130,671	119,384–148,294	108,454–116,478	123,051–141,971	118,187–126,765
		129,059.2	131,410.1	111,817.5	131,932.4	120,792.6
		± 1151.89	± 9419.46	± 2222.63	± 6406.92	± 2495.34
Mean neighborhood population age		42.51–43.38	44.19–48.86	38.56–39.40	42.47–44.43	39.61–40.71
		42.93	46.68	38.97	43.59	39.93
		± 0.28	± 1.68	± 0.22	± 0.68	± 0.38

For each variable, the table shows range, mean, and standard deviation, calculated across the ten subsets. The totals per category may not be equal to 10,000 due to neighborless individuals who were excluded from the Local Moran’s analysis.

### Geographic footprint of YPLLG

**Figure 1:**
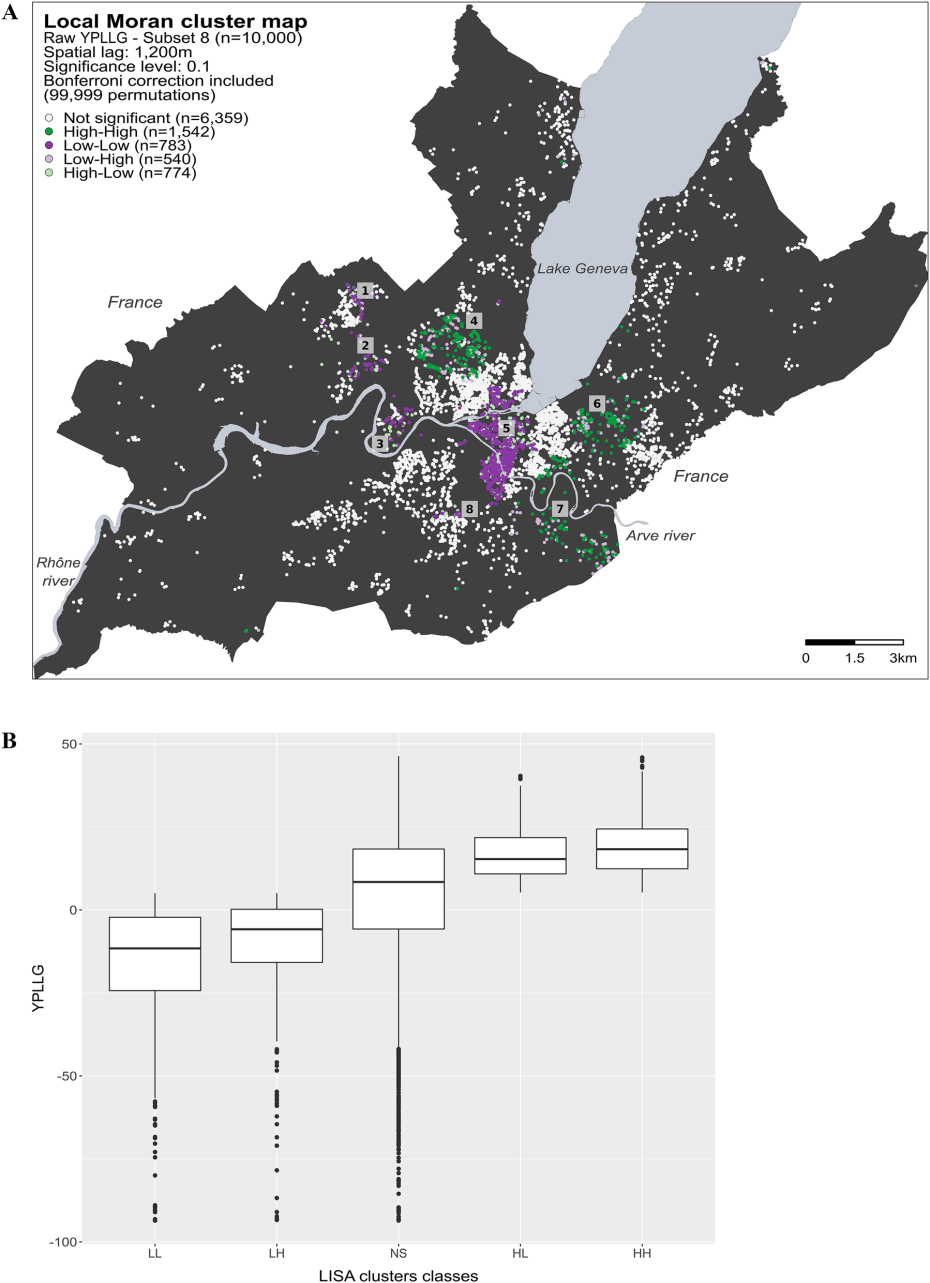
Spatial footprint of YPLLG for the raw model. (**A**) Local Moran cluster map was calculated for a random subset of 10,000 individuals taken from the 22,751 individuals in the state of Geneva for the 2009–2016 period (Subset 8). White dots represent individuals without spatial dependence (i.e., those whose Local Moran’s I p-values adjusted with Bonferroni are not significant). Dark green dots (High–High cluster) represent individuals with high YPLLG values (i.e., those that lived longer than expected) surrounded by individuals with similar YPLLG values within a distance of 1200 m. Dark purple dots (Low–Low cluster) represent individuals with low YPLLG values (i.e., those that lived shorter than expected) surrounded by individuals with similar YPLLG values. Light purple dots (Low–High spatial outliers) represent individuals with high YPLLG values surrounded by individuals with low YPLLG values, and light green dots (High–Low spatial outliers) represent individuals with low YPLLG values surrounded by individuals with high YPLLG values. Indicative landmarks are shown on the map to facilitate the interpretation of the results (#1–#8). [Source (administrative boundaries): https://www.swisstopo.admin.ch/, 2020; the map was produced using R, package ggplot version 3.3.5.]. (**B**) Distribution of YPLLG values for each Local Moran cluster type (*LL* Low–Low, *LH* Low–High, *NS* not significant, *HL* High–Low, *HH* High–High).

**Figure 2:**
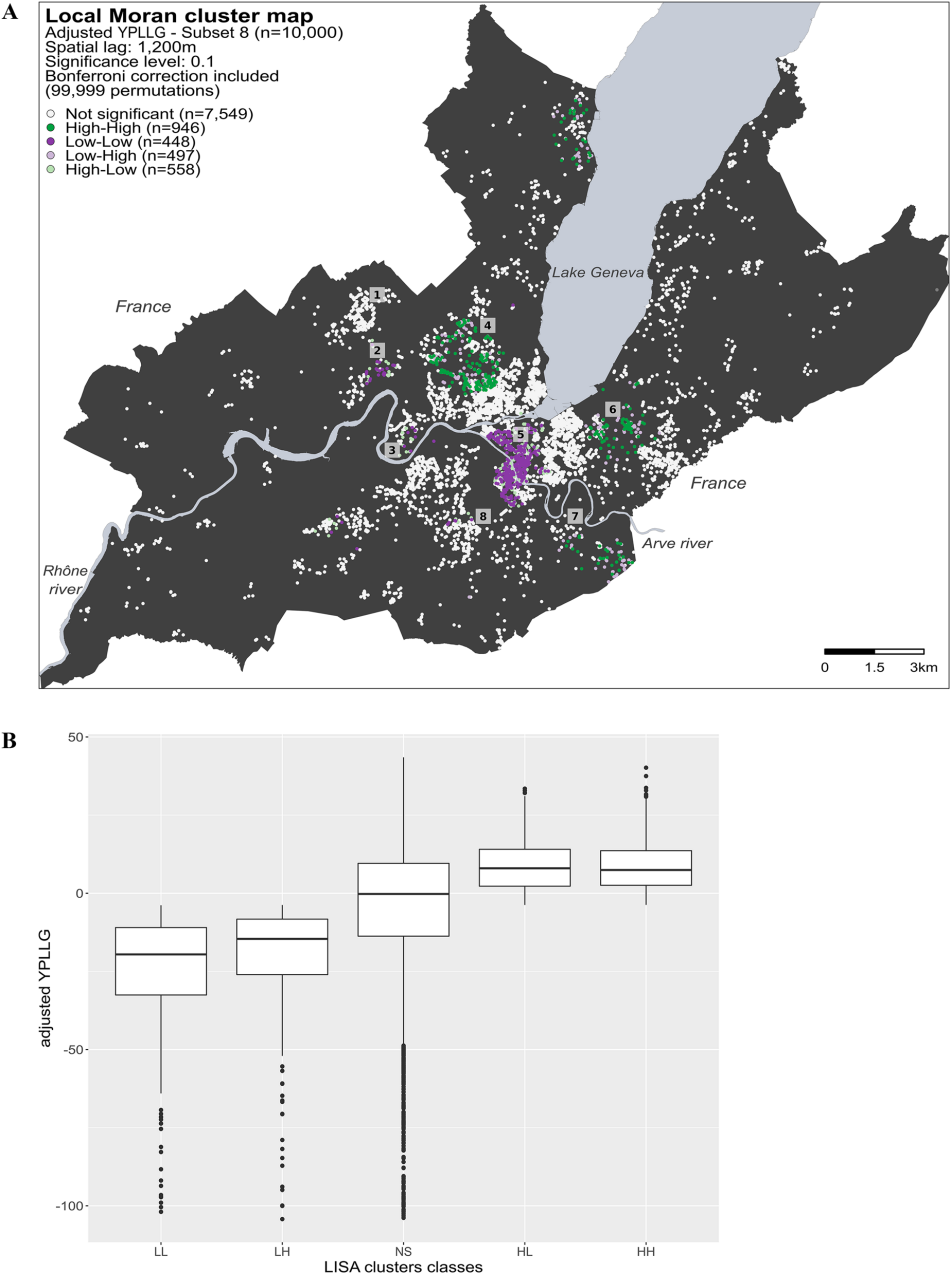
Spatial footprint of YPLLG for the adjusted model. (**A**) Local Moran cluster map was calculated for a random subset of 10,000 individuals taken from the 22,751 individuals in the state of Geneva for the 2009–2016 period (Subset 8). White dots represent individuals without spatial dependence (i.e., those whose Local Moran’s I p-values adjusted with Bonferroni are not significant). Dark green dots (High–High cluster) represent individuals with high adjusted YPLLG values (i.e., those that that lived longer than expected) surrounded by individuals with similar values within a distance of 1200 m. Dark purple dots (Low–Low cluster) represent individuals with low adjusted YPLLG values (i.e., those that lived shorter than expected) surrounded by individuals with similar values. Light purple dots (Low–High spatial outliers) represent individuals with high adjusted YPLLG values surrounded by individuals with low adjusted YPLLG values, and light green dots (High–Low spatial outliers) represent individuals with low adjusted YPLLG values surrounded by individuals with high adjusted YPLLG values. Indicative landmarks are shown on the map to facilitate the interpretation of the results (#1–#8). [Source (administrative boundaries): https://www.swisstopo.admin.ch/, 2020; the map was produced using R, package ggplot version 3.3.5.]. (**B**) Distribution of adjusted YPLLG values for each Local Moran cluster type (*LL* Low–Low, *LH* Low–High, *NS* not significant, *HL* High–Low, *HH* High–High).

We identified the type of spatial association undergoing at each location by comparing the YPLLG value of an individual with the mean YPLLG value of its neighbors, illustrated on the Local Moran cluster maps for the raw and the adjusted YPLLG models (Figs. 1A, 2A). On the maps, white dots represent locations showing no spatial dependence, dark green dots (High–High) and dark purple dots (Low–Low) represent individuals with high YPLLG, respectively low YPLLG, surrounded by individuals with similar values. Light purple dots (Low–High) and light green dots (High–Low) represent discordant behaviors (spatial outliers), where an individual’s YPLLG value differs considerably from the mean YPLLG value of its neighbors.

Because similar geographic footprints were obtained for the ten subsets, in this section, we only refer to the Local Moran cluster map from subset 8. Results from the other subsets are available in the Supplementary Materials (Figs. S2, S3).

Analysis revealed a clear spatial structure for the raw YPLLG model in the state of Geneva (Fig. 1A). Low–Low clusters were mostly concentrated downtown and in the west areas (landmarks #1-#3, #5, #8), while High–High clusters were located in the south and north areas (landmarks #4, #6, #7). The distribution of YPLLG values within each cluster type (Fig. 1B) showed significant differences between clusters of low and high YPLLG values (mean difference of 34.93 years, p < 0.001).

After controlling for nationality, neighborhood income, and median neighborhood age, we obtained a similar geographic footprint of YPLLG values (Fig. 2A), with a moderate reduction in the size of low (43%) and high (39%) YPLLG cluster footprints. The adjustment thinned down the low YPLLG clusters in landmarks #2, #3, and #5, while the Low–Low cluster in landmark #3 has disappeared. For high YPLLG clusters, the reduction mainly affects clusters located in landmarks #4 and #7. The difference in YPLLG values between individuals in clusters of low and high YPLLG (Fig. 2B) was also considerable (mean YPLLG difference of 32.93 years, p < 0.001).

## Discussion

In using spatial cluster detection methods on individual georeferenced mortality data, our analyses revealed a clear spatial structure of YPLLG across the state of Geneva. We found that individuals living shorter than expected (i.e., with low YPLLG values) were clustered in downtown and western areas (landmarks #1–#3, #5, #8 in Figs. 1A, 2A), while individuals living longer than expected (i.e., with high YPLLG values) were clustered in the southern and in the northern areas of the state (landmarks #4, #6, #7 in Figs. 1A, 2A). In addition, we observed demographic and socioeconomic differences between low YPLLG clusters (Low–Low) and high YPLLG clusters (High–High), where the latter population was characterized by a higher proportion of non-Swiss and men who were living in more poorer and younger neighborhoods. Adjusting for individual-level and neighborhood-level covariates moderately mitigated the size of YPLLG clusters (43% reduction for Low–Low clusters and 39% reduction for High–High clusters). While this thinned down most of the spatial clusters detected in Fig. 1A, the geographic footprint of YPLLG remained, suggesting that population demographics and socioeconomic status might not fully explain patterns in mortality across the state of Geneva.

The significant association between YPLLG and neighborhood median income is consistent with results from other studies, where a relationship between mortality and the socioeconomic position in small geographic areas are also observed^24–26,43^. These studies suggest interconnected pathways between social and health inequalities. Unlike Buajitti et al.^23^, we did not find a higher proportion of foreigners in areas of lower premature mortality, which may contradict the immigrant health advantage described elsewhere^44^. However, the literature also states that this advantage is mitigated over time^45^. Therefore, indicators using a general age cutoff (e.g., 75 years) may not reflect the entire lifespan of individuals and overestimate the immigrant health advantage. Furthermore, our analysis was conducted on individual-level data rather than at an aggregated scale which might exacerbate these differences. The higher proportion of foreigners found in low YPLLG clusters could partially be explained by the fact that we assigned to individuals the Life Expectancy at Birth from Swiss life tables, which is among the highest in the world^12^. Hence, it could overestimate the LEB of non-Swiss people and, thus, the absolute value of YPLLG. It may also indicate unequal situations between Swiss nationals and migrants as foreigners face worse living conditions and quality of life in Switzerland^46^. This is consistent with other studies highlighting associations between deprived life conditions and higher mortality rate^3,47–49^. Further epidemiological studies are necessary to disentangle the underlying factors leading to the geographic footprints of life expectancy inequalities discovered in this study.

Interestingly, we identified a few similarities between the spatial patterns of health inequalities revealed by YPLLG and those detected in another study assessing the spatial dependence of body mass index (BMI) in Geneva^36^. Indeed, some clusters of elevated BMI overlap clusters of low YPLLG and conversely (data not shown). Thus, we may presume that both outcomes are spatially interlinked and that some of these premature deaths may be related to conditions associated with a high BMI, such as cardiovascular disease and diabetes.

When comparing YPLLG and YPLL values among cluster types, we observed similar trends between both indicators, showing that they measure comparable mortality inequalities. For instance, High–High clusters present a YPLL value of 0, while Low–Low clusters have a positive YPLL mean value of 12 in both raw and adjusted models. However, due to the nature of YPLL that only identifies subjects that faced premature death, YPLLG may constitute a more appropriate indicator to measure health inequalities.

### Strengths

As far as we know, this is the first study that analyzes spatial dependence of a life expectancy indicator using a large sample size of individual-level mortality data (n = 22,751), making it possible to identify small areas inequalities in health. The fact that no significant differences were detected in the geographic footprint of YPLLG between the ten random subsets demonstrates that our results are not specific to our dataset, and that pattern of life expectancy inequalities are deeply embedded in the territory of the Geneva state.

### Limitations

Several important variables were not available in the original raw dataset, including socioeconomic status, cause of death, and prevalence of comorbidities. In addition, we removed 1611 individuals (5.8% of the original dataset) for whom gender could not be assigned using the name-to-gender inference, which could lead to underestimating the proportion of foreigners in the final dataset^30^. However, we did not notice any trend in the missing cases when we compared the number of deaths included in our study (stratified by gender and nationality) with those published by the Cantonal Statistical Office at the municipality level (Supplementary Table S4). Additional limitations may also originate from the fact that only married couples could be included in the calculation of household income, and we only included the last place of residence of individuals, which may not represent where they spent most of their lives. Furthermore, our results may only be representative of the period included in the analysis (2009–2016).

### Policy implications

Switzerland, and in particular the state of Geneva, has one of the highest LEB and quality of life worldwide. However, this does not prevent the state from presenting considerable health inequalities. Contrasts in socioeconomic conditions like nationality and neighborhood income result in profound geographic disparities and reveal deprived living environment areas. The same areas were by the way recently shown to be exposed to SARS-CoV-2 clusters that persisted significantly longer than elsewhere in the state^50^. However, one can also consider the existence of such spatial structures as opportunities for intervention. The present findings should encourage authorities to acknowledge geographic areas facing health inequalities and to favor, in these zones, the development of adequate public health policies to create conditions of more equitable living environments. Such policies should consider the social component rather than focusing exclusively on treating risk factors^6,51^. For instance, decision-makers could: (i) favor the development of urban districts socioeconomically mixed; (ii) improve living conditions of neighborhoods with a high rate of foreigners; (iii) favor access to health in local areas where exist higher prevalence of chronic diseases or; (iv) allocating economic assistance in elderly.

## Conclusion

Individual-based spatial patterns of life expectancy translate health inequalities footprints on a territory. Our study revealed specific spatial patterns of YPLLG in the state of Geneva using spatial cluster detection methods on individual georeferenced mortality data. The proportion of women, the proportion of Swiss, and the median neighborhood income were significantly lower for populations in low YPLLG clusters than for populations in high YPLLG clusters. Adjustment for nationality and neighborhood income slightly reduced the footprint of low YPLLG clusters but did not modify the population characteristics within clusters. Results highlight the worth of precision public health relying on spatial methods to assess health inequalities at a local level and target vulnerable populations.

## Supplementary Information

Supplementary Information.

## Data Availability

Datasets used in the current study are available at https://zenodo.org/badge/latestdoi/418981695.

## Code availability

All code for data cleaning and analysis associated with the current submission is available at https://zenodo.org/badge/latestdoi/418981695.
