# Supplementary material for: Geographic footprints of life expectancy inequalities in the state of Geneva, Switzerland
Source: Sci Rep. 2021 Dec 2;11:23326. doi: 10.1038/s41598-021-02733-x (PMC8639743; doi:10.1038/s41598-021-02733-x)
Supplement: Supplementary file 1 — Supplementary Information. [file 41598_2021_2733_MOESM1_ESM.pdf]

Geographic footprint of life expectancy inequalities in the state of Geneva, Switzerland - Supplementary Materials

Anaïs Ladoy[1,2], Juan R Vallarta-Robledo[2,3,4], David De Ridder[1,2,3,4], José Luis Sandoval[2,5,6], Silvia Stringhini[2,3,4,7], Henrique DaCosta[8], Idris Guessous[2,3,6], and Stéphane Joost[1,2,4,9]

[1]Laboratory of Geographic Information Systems (LASIG), School of Architecture, Civil and Environmental Engineering (ENAC), Ecole Polytechnique Fédérale de Lausanne (EPFL), Lausanne, Switzerland
[2]Group of Geographic Information Research and Analysis in Population Health (GIRAPH), Geneva, Switzerland
[3]Faculty of Medicine, University of Geneva, Geneva, Switzerland
[4]Unit of Population Epidemiology, Department of Primary Care, Geneva University Hospitals, Geneva, Switzerland
[5]Department of Oncology, Geneva University Hospitals, Geneva, Switzerland
[6]Division of Primary Care Medicine, Department of Primary Care, Geneva University Hospitals, Geneva, Switzerland
[7]University Centre for General Medicine and Public Health, University of Lausanne, Lausanne, Switzerland
[8]Réseau Delta, HMO, Geneva, Switzerland
[9]La Source School of Nursing, University of Applied Sciences and Arts Western Switzerland (HES-SO), Lausanne, Switzerland

Corresponding author: Stéphane Joost. Laboratory of Geographic Information Systems (LASIG), School of Architecture, Civil and Environmental Engineering (ENAC), Ecole Polytechnique Fédérale de Lausanne (EPFL), Lausanne, Switzerland. email: stephane.joost@epfl.ch, tel:41-21-693-57-82

Figure S1: Distribution of Years of Potential Life Lost or Gained (YPLLG) for the 22,751 deceased included in our study (2009-2016)

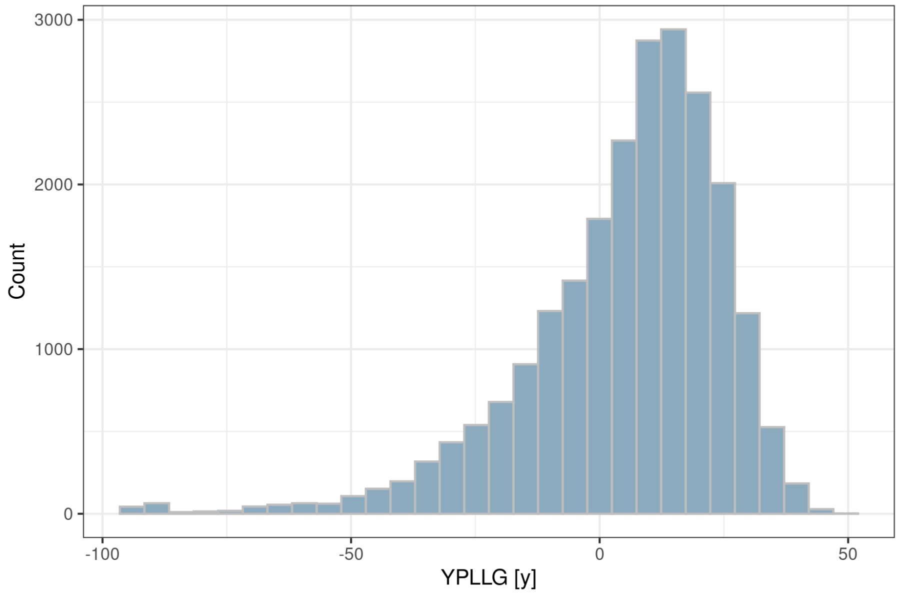

Equation S1: Estimation of adjusted Years of Potential Life Lost or Gained (YPLLG) values

In our study, we performed the same spatial analysis on both raw YPLLG and adjusted YPLLG values.
The objective of this was to evaluate the degree to which spatial clusters of YPLLG were explained by the spatial distribution of income and age distribution, while also taking into account potential individual-level confounders such as nationality. (sex and gender are already included in the attribution of LEB).

We obtained adjusted estimates of YPLLG using a median regression. This regression model is preferred to linear regression when the dependant variable is highly skewed, as it is in our case (see the distribution of YPLLG in Figure S1) [2]. As the name suggests, the median regression differs from the linear regression model by estimating the median of the dependant variable (here YPLGG) rather than the mean. Regression parameters in the median regression model are then obtained by minimizing the sum of the absolute value of residuals instead of minimizing the sum of squares of the residuals.

The median regression model is defined as follows:

$$Y P\hat{L}LG = \hat{\beta}_0 + \hat{\beta}_1 \overline{swiss} + \hat{\beta}_2 \overline{income} + \hat{\beta}_3 \overline{pop.age} \tag{1}$$

Analysis was conducted using the R package *quantreg* (version 5.86)[1].

Table S1: Sample characteristics of the 10 random subsets. As the subsets have 10,000 observations, the proportion of women and foreigners can be deducted by multiplying the percentage values of these groups by 1,000.

**Subset**	**1**	**2**	**3**	**4**	**5**	**6**	**7**	**8**	**9**	**10**
Women [%]	53.1	53.3	53.2	53.3	53.8	52.7	53.0	53.5	53.2	52.6
Foreigners [%]	20.5	20.6	20.5	20.1	20.8	20.7	20.3	20.2	20.5	20.1
YPLLG [y]	5.3 (±20.1)	5.1 (±20.1)	5.0 (±20.4)	5.1 (±20.1)	5.5 (±19.9)	5.3 (±20.1)	5.2 (±20.0)	5.3 (±20.2)	5.0 (±20.2)	5.0 (±20.1)
Age at death [y]	79.5 (±15.3)	79.4 (±15.4)	79.3 (±15.6)	79.4 (±15.4)	79.7 (±15.1)	79.4 (±15.3)	79.4 (±15.2)	79.5 (±15.4)	79.3 (±15.4)	79.3 (±15.3)
Neighborhood median household income [CHF]	128211 (±41108)	127893 (±40424)	128105 (±41208)	128318 (±41135)	127924 (±41195)	128278 (±41449)	128295 (±40987)	128312 (±41335)	127954 (±40746)	127971 (±41031)
Neighborhood population median age [y]	43.2 (±10.0)	43.0 (±9.7)	42.8 (±9.5)	42.9 (±9.7)	43.0 (±9.8)	43.0 (±9.8)	42.9 (±9.6)	43.0 (±9.6)	43.0 (±9.8)	42.9 (±9.7)

Table S2: Spatial weight characteristics of the 10 random subsets.

**Subset**	**1**	**2**	**3**	**4**	**5**	**6**	**7**	**8**	**9**	**10**
Mean number of neighbors	758.6	737.6	756.9	771.5	767.1	752.3	745.7	768.8	749.3	761.0
Neighborless individuals	1	2	4	2	2	2	0	2	3	4

Table S3: Median regression values for the 10 random subsets, with p-values show in brackets

**Subset Dependant variable**	**1**	**2**	**3**	**4**	**5**	**6**	**7**	**8**	**9**	**10**
Constant	-9.78 (p<0.001)	-11.95 (p<0.001)	-11.26 (p<0.001)	-11.68 (p<0.001)	-10.56 (p<0.001)	-11.84 (p<0.001)	-12.99 (p<0.001)	-10.56 (p<0.001)	-11.51 (p<0.001)	-10.96 (p<0.001)
Nationality (Swiss)	6.61 (p<0.001)	6.70 (p<0.001)	6.60 (p<0.001)	6.25 (p<0.001)	6.79 (p<0.001)	6.76 (p<0.001)	6.54 (p<0.001)	5.86 (p<0.001)	6.51 (p<0.001)	6.13 (p<0.001)
Neighborhood median housing income	2e-5 (p<0.001)	3e-5 (p<0.001)	3e-5 (p<0.001)	3e-5 (p<0.001)	2e-5 (p<0.001)	3e-5 (p<0.001)	3e-5 (p<0.001)	3e-5 (p<0.001)	3e-5 (p<0.001)	3e-5 (p<0.001)
Neighborhood population median age	0.25 (p<0.001)	0.28 (p<0.001)	0.26 (p<0.001)	0.27 (p<0.001)	0.26 (p<0.001)	0.27 (p<0.001)	0.28 (p<0.001)	0.26 (p<0.001)	0.27 (p<0.001)	0.26 (p<0.001)

Figure S2: Spatial footprint of YPLLG for the raw model. Local Moran cluster maps were calculated for ten random subsets of 10,000 individuals taken from the 22,751 individuals in the state of Geneva for the 2009-2016 period. White dots represent individuals without spatial dependence (i.e., those whose Local Moran's I p-values adjusted with Bonferroni are not significant). Dark green dots (High-High cluster) represent individuals with high YPLLG values (i.e., those that lived longer than expected) surrounded by individuals with similar YPLLG values within a distance of 1,200m. Dark purple dots (Low-Low cluster) represent individuals with low YPLLG values (i.e., those that lived shorter than expected) surrounded by individuals with similar YPLLG values. Light purple dots (Low-High spatial outliers) represent individuals with high YPLLG values surrounded by individuals with low YPLLG values, and light green dots (High-Low spatial outliers) represent individuals with low YPLLG values surrounded by individuals with high YPLLG values. [Source (administrative boundaries): https://www.swisstopo.admin.ch/, 2020; the maps were produced using R, package ggplot version 3.3.5.]

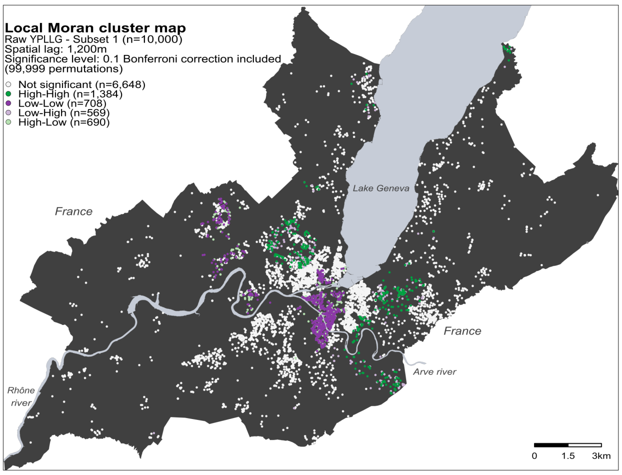

(a) Subset 1

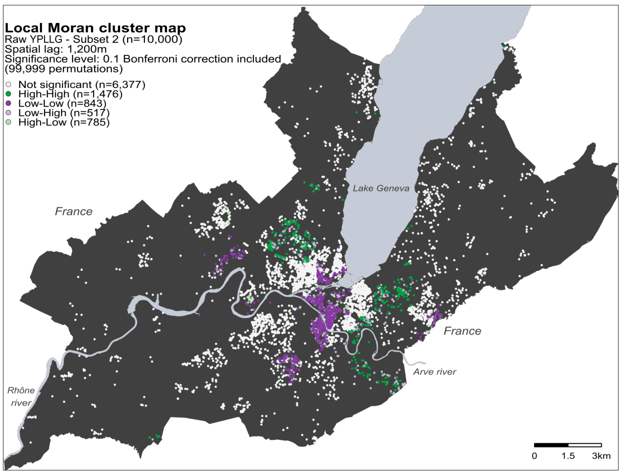

(b) Subset 2

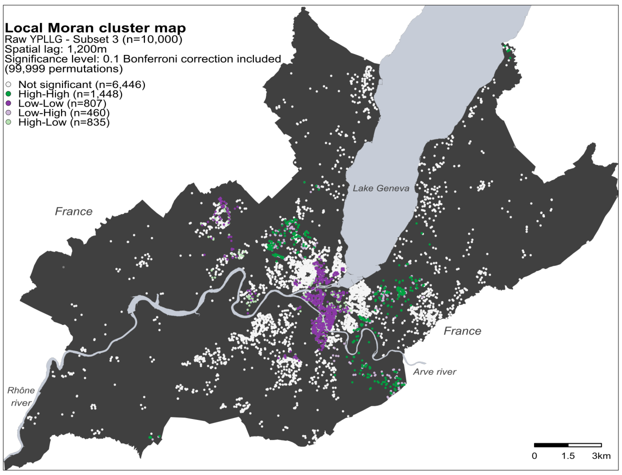

(c) Subset 3

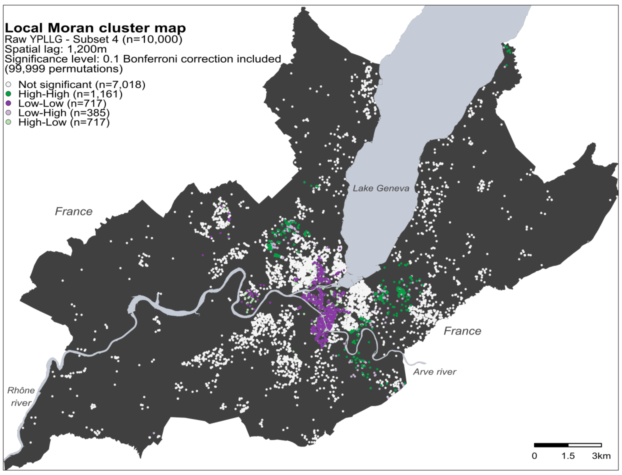

(d) Subset 4

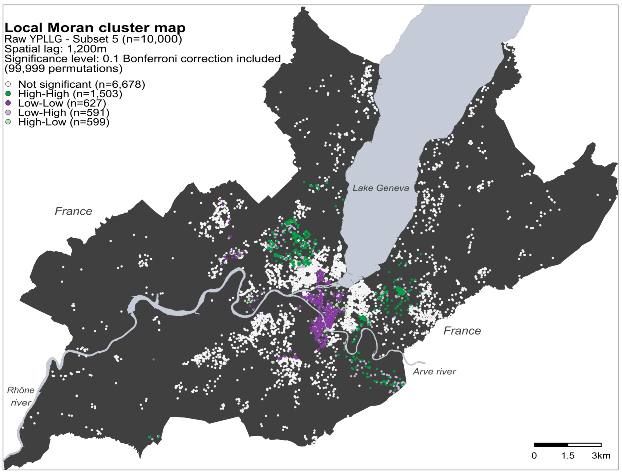

(e) Subset 5

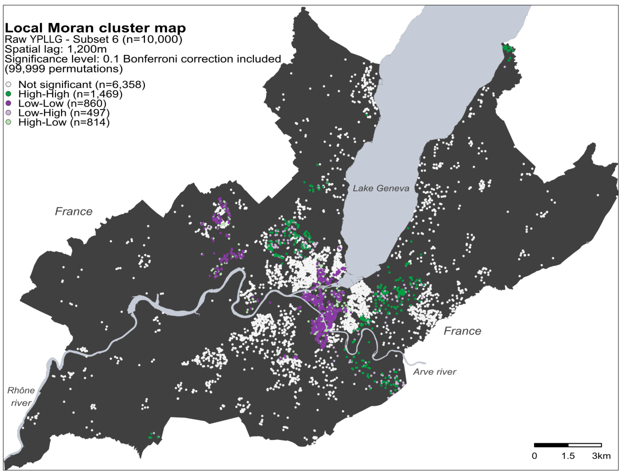

(f) Subset 6

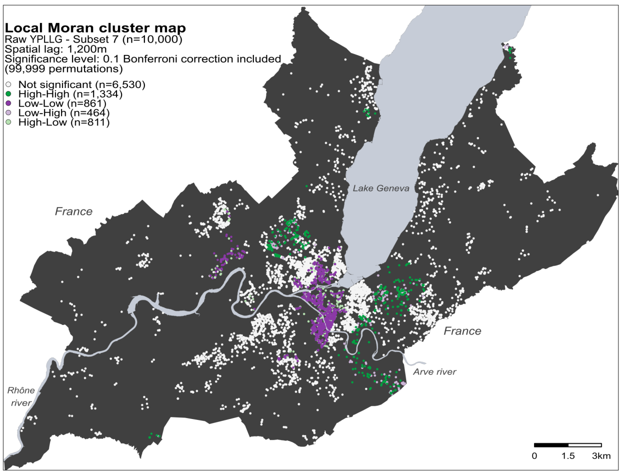

(g) Subset 7

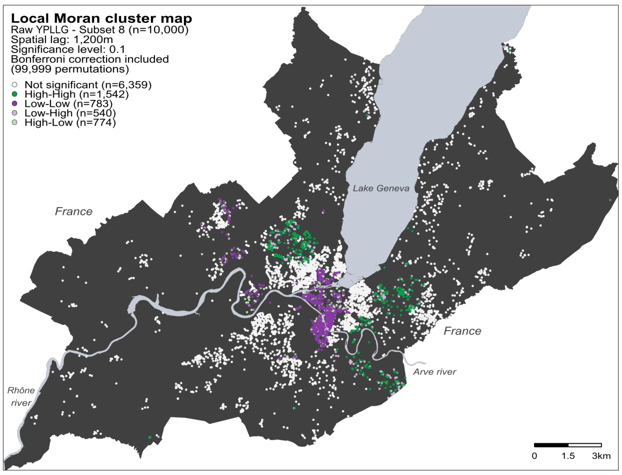

(h) Subset 8

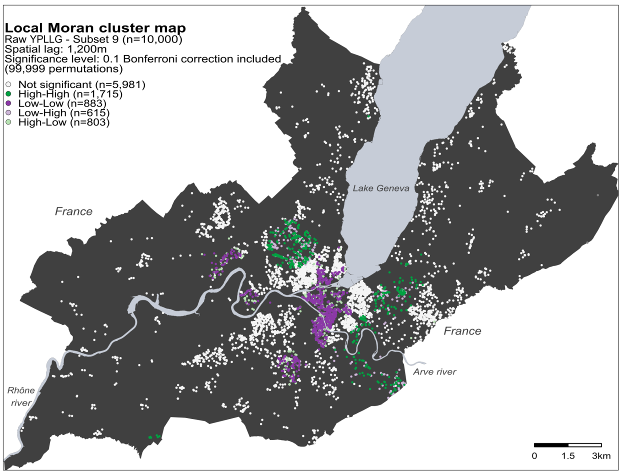

(i) Subset 9

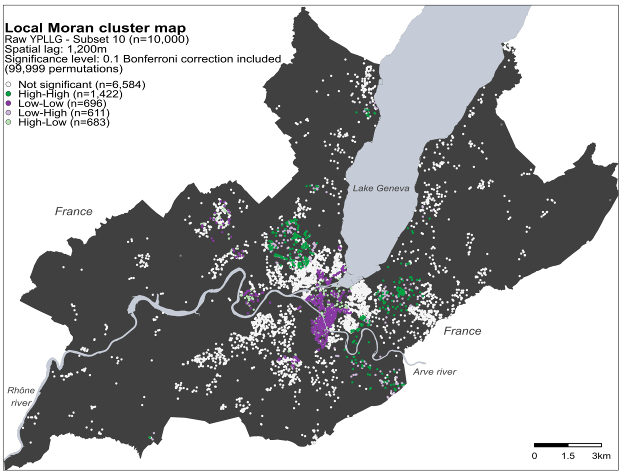

(j) Subset 10

Figure S3: Spatial footprint of YPLLG for the adjusted model. Local Moran cluster maps were calculated for ten random subsets of 10,000 individuals taken from the 22,751 individuals in the state of Geneva for the 2009-2016 period. White dots represent individuals without spatial dependence (i.e., those whose Local Moran's I p-values adjusted with Bonferroni are not significant). Dark green dots (High-High cluster) represent individuals with high YPLLG values (i.e., those that lived longer than expected) surrounded by individuals with similar YPLLG values within a distance of 1,200m. Dark purple dots (Low-Low cluster) represent individuals with low YPLLG values (i.e., those that lived shorter than expected) surrounded by individuals with similar YPLLG values. Light purple dots (Low-High spatial outliers) represent individuals with high YPLLG values surrounded by individuals with low YPLLG values, and light green dots (High-Low spatial outliers) represent individuals with low YPLLG values surrounded by individuals with high YPLLG values. [Source (administrative boundaries): https://www.swisstopo.admin.ch/, 2020; the maps were produced using R, package ggplot version 3.3.5.]

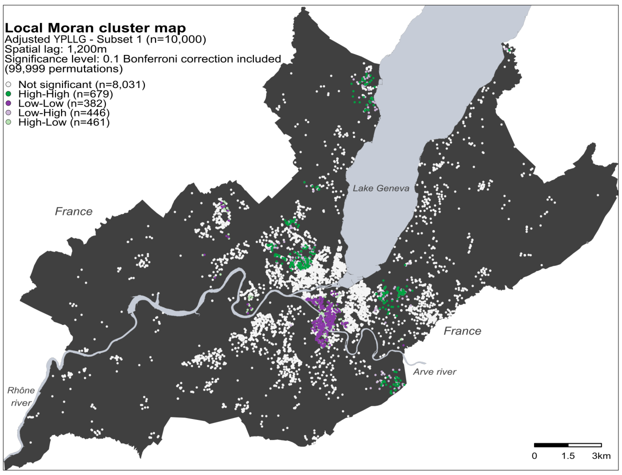

(a) Subset 1

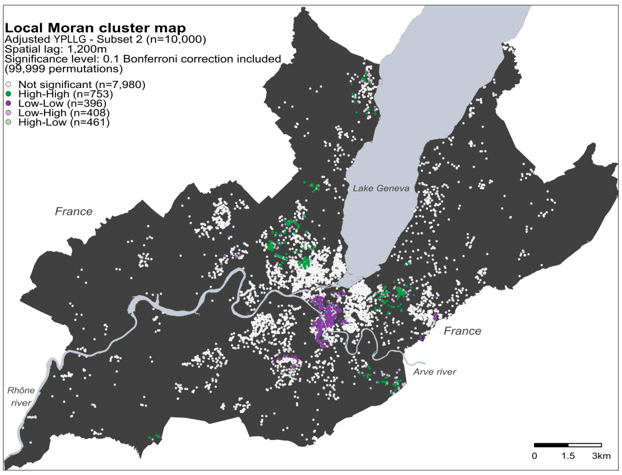

(b) Subset 2

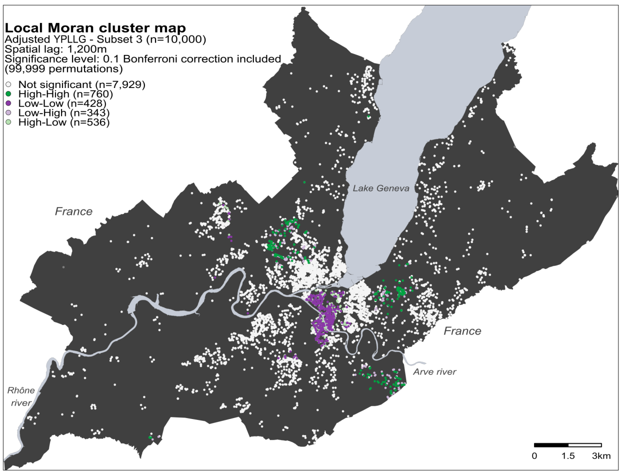

(c) Subset 3

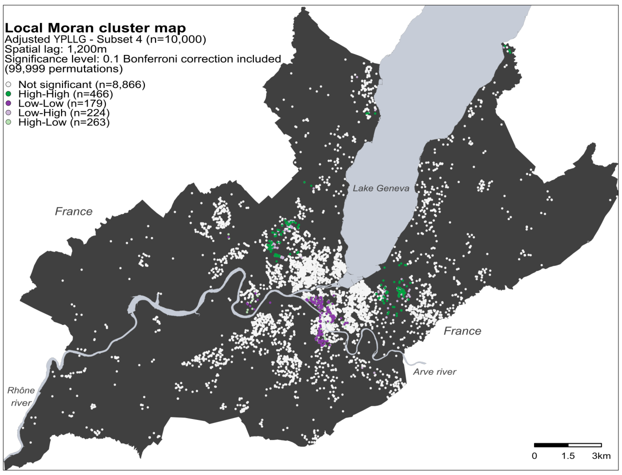

(d) Subset 4

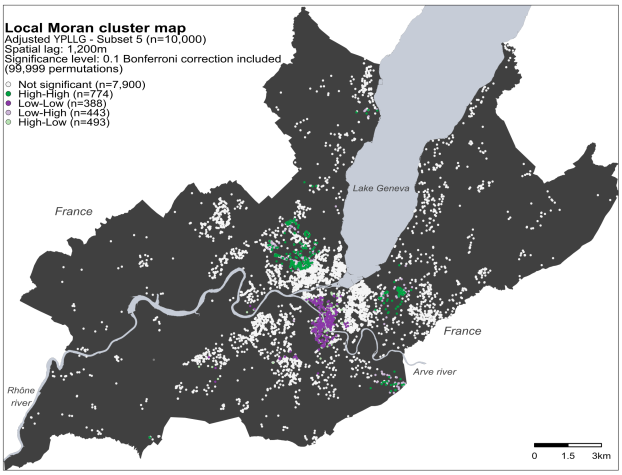

(e) Subset 5

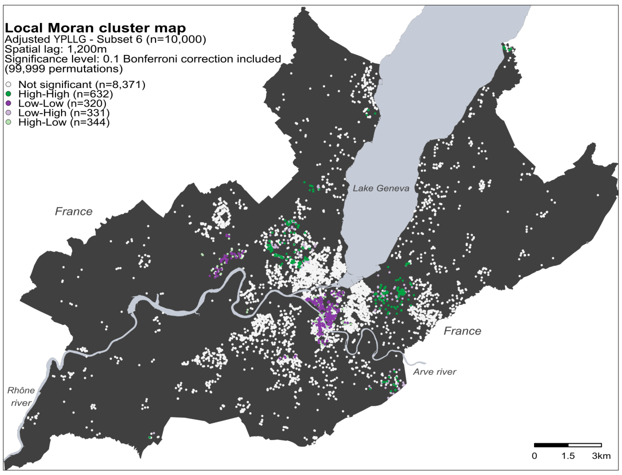

(f) Subset 6

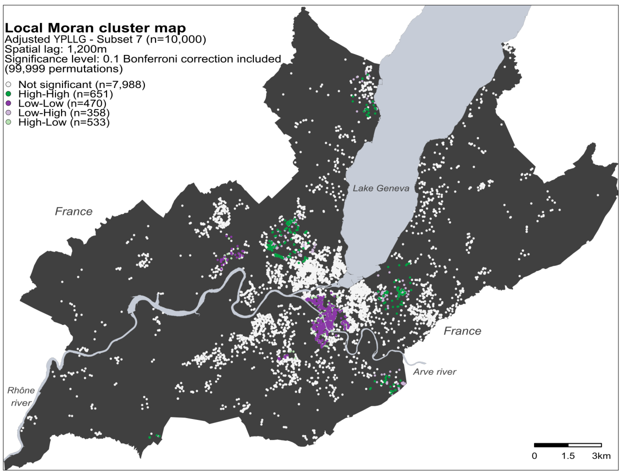

(g) Subset 7

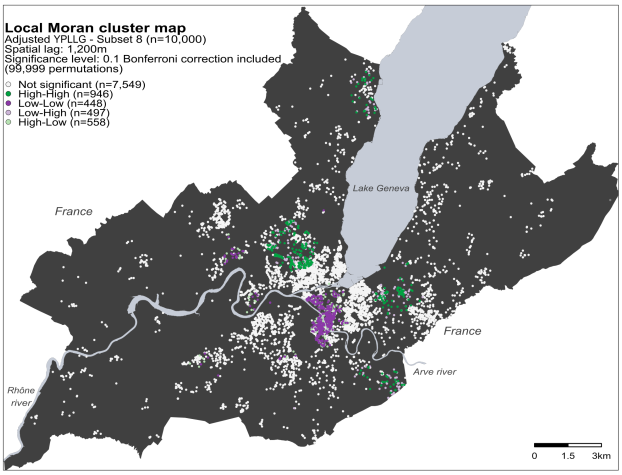

(h) Subset 8

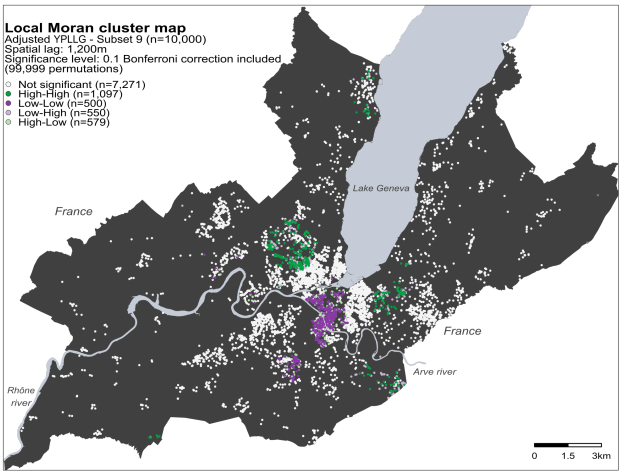

(i) Subset 9

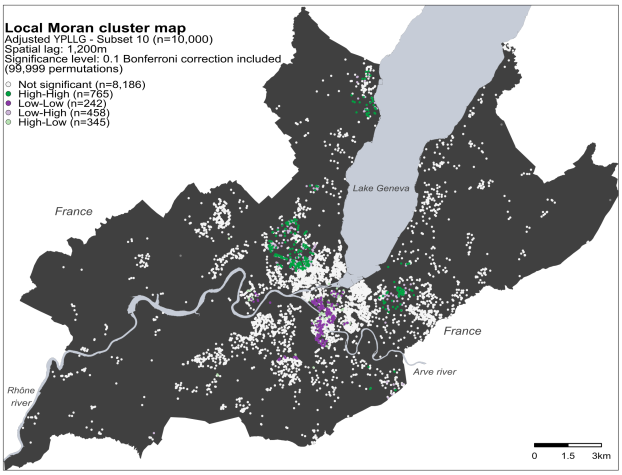

(j) Subset 10

Table S4: Comparison of the number of deaths published by the Federal Statistical Office (*FSO*) of Switzerland with the number included in our study (*S*), partitioned by municipality, sex and nationality. The highlighted cells correspond to positive differences, i.e. individuals missing for the specific year/municipality combination. The proportion of missing individuals correlates with the size of municipalities, with no specific exclusion pattern between year, gender, or nationality. A negligible fraction of the differences are negative, indicating that for the specific year/municipality combination, the number of deceased included in our study was larger than the deaths recorded by the Statistical Office. This can be explained by errors such as inaccurate name-to-gender inference, inaccurate data, or geocoding errors.

			2009			2010			2011			2012			2013			2014			2015			2016		
Municipality	Gender	Natl.	FSO	S	Δ	FSO	S	Δ	FSO	S	Δ	FSO	S	Δ	FSO	S	Δ	FSO	S	Δ	FSO	S	Δ	FSO	S	Δ
Aire-la-Ville	Female	Non Swiss	0	0	0	0	0	0	0	0	0	0	0	0	0	0	0	0	0	0	0	0	0	0	0	0
		Swiss	0	0	0	1	1	0	0	0	0	0	0	0	0	0	0	1	1	0	2	2	0	2	2	0
	Male	Non Swiss	0	0	0	0	0	0	0	0	0	0	0	0	0	0	0	0	0	0	0	0	0	1	1	0
		Swiss	1	1	0	2	2	0	1	0	1	1	1	0	1	2	-1	3	3	0	5	5	0	2	2	0
Anières	Female	Non Swiss	2	2	0	3	3	0	1	1	0	1	1	0	2	1	1	0	0	0	0	0	0	0	0	0
		Swiss	9	7	2	17	15	2	12	12	0	2	2	0	6	6	0	2	2	0	0	1	-1	10	9	1
	Male	Non Swiss	1	0	1	3	1	2	2	2	0	2	2	0	0	0	0	1	0	1	1	1	0	0	0	0
		Swiss	8	8	0	4	4	0	6	5	1	1	0	1	4	4	0	6	3	3	4	2	2	6	7	-1
Avully	Female	Non Swiss	0	0	0	0	0	0	0	0	0	0	0	0	1	1	0	0	0	0	1	0	1	1	1	0
		Swiss	1	1	0	7	8	-1	4	4	0	2	2	0	1	1	0	0	0	0	1	1	0	3	3	0
	Male	Non Swiss	0	0	0	0	0	0	0	0	0	1	1	0	0	1	-1	0	0	0	0	0	0	1	1	0
		Swiss	2	2	0	2	2	0	3	3	0	5	4	1	3	3	0	3	3	0	3	3	0	2	2	0
Avusy	Female	Non Swiss	0	0	0	0	0	0	0	0	0	0	0	0	1	1	0	0	0	0	0	0	0	0	0	0
		Swiss	1	1	0	1	1	0	3	2	1	1	1	0	3	2	1	0	0	0	2	2	0	1	1	0
	Male	Non Swiss	0	0	0	0	0	0	1	1	0	0	0	0	0	0	0	1	1	0	0	0	0	0	0	0
		Swiss	1	1	0	4	4	0	2	2	0	4	4	0	2	2	0	2	2	0	1	1	0	4	4	0
Bardonnex	Female	Non Swiss	0	0	0	0	0	0	1	1	0	0	0	0	2	2	0	1	1	0	0	0	0	0	0	0
		Swiss	2	1	1	5	5	0	2	2	0	3	3	0	2	4	-2	4	3	1	1	1	0	3	2	1
	Male	Non Swiss	0	0	0	0	0	0	2	2	0	1	1	0	0	0	0	1	1	0	1	1	0	1	0	1
		Swiss	6	5	1	3	3	0	3	2	1	9	8	1	5	3	2	6	6	0	5	5	0	4	4	0
Bellevue	Female	Non Swiss	1	1	0	1	0	1	0	0	0	1	1	0	0	0	0	2	0	2	0	0	0	2	2	0
		Swiss	4	3	1	3	2	1	5	3	2	1	1	0	0	0	0	3	3	0	2	2	0	0	0	0
	Male	Non Swiss	0	0	0	0	0	0	1	1	0	1	1	0	1	1	0	1	0	1	2	1	1	0	0	0
		Swiss	2	0	2	1	2	-1	4	2	2	2	2	0	5	4	1	3	3	0	4	4	0	5	5	0
Bernex	Female	Non Swiss	3	2	1	4	3	1	2	3	-1	4	4	0	2	2	0	0	0	0	5	4	1	7	6	1
		Swiss	38	36	2	20	17	3	16	15	1	21	19	2	30	27	3	19	16	3	23	19	4	28	26	2
	Male	Non Swiss	6	7	-1	4	3	1	5	3	2	7	8	-1	6	7	-1	7	5	2	6	5	1	6	5	1
		Swiss	29	26	3	29	29	0	26	22	4	24	20	4	26	24	2	19	17	2	34	28	6	22	18	4
Carouge	Female	Non Swiss	11	9	2	15	13	2	7	6	1	19	17	2	10	10	0	12	11	1	16	14	2	17	10	7
		Swiss	64	60	4	58	54	4	61	59	2	65	54	11	42	42	0	70	61	9	44	34	10	56	50	6
	Male	Non Swiss	17	17	0	11	10	1	15	14	1	13	14	-1	15	16	-1	18	12	6	19	17	2	18	17	1
		Swiss	59	50	9	52	45	7	41	39	2	45	37	8	39	32	7	61	50	11	54	45	9	50	44	6
Cartigny	Female	Non Swiss	1	1	0	0	0	0	0	0	0	0	0	0	0	0	0	0	0	0	0	0	0	0	0	0
		Swiss	0	0	0	4	3	1	1	1	0	2	2	0	2	2	0	1	1	0	0	0	0	0	0	0
	Male	Non Swiss	0	0	0	0	0	0	0	0	0	0	0	0	0	0	0	0	0	0	0	0	0	2	2	0
		Swiss	3	1	2	0	0	0	1	1	0	2	1	1	2	1	1	4	4	0	2	2	0	3	3	0

Municipality	Gender	Natl.	2009 FSO	2009 S	2009 Δ	2010 FSO	2010 S	2010 Δ	2011 FSO	2011 S	2011 Δ	2012 FSO	2012 S	2012 Δ	2013 FSO	2013 S	2013 Δ	2014 FSO	2014 S	2014 Δ	2015 FSO	2015 S	2015 Δ	2016 FSO	2016 S	2016 Δ
Chancy	Female	Non Swiss	0	0	0	0	0	0	0	0	0	1	0	1	0	0	0	0	0	0	0	0	0	0	0	0
		Swiss	1	1	0	1	0	1	0	0	0	3	3	0	2	2	0	2	2	0	2	2	0	1	1	0
	Male	Non Swiss	0	0	0	0	0	0	0	0	0	0	0	0	0	0	0	0	0	0	0	0	0	0	0	0
		Swiss	2	2	0	1	1	0	0	0	0	4	4	0	1	1	0	1	1	0	1	1	0	3	3	0
Choulex	Female	Non Swiss	0	0	0	0	0	0	0	0	0	0	0	0	0	0	0	0	0	0	0	0	0	0	0	0
		Swiss	6	5	1	9	9	0	8	7	1	3	3	0	3	5	-2	2	2	0	5	5	0	3	3	0
	Male	Non Swiss	0	0	0	0	1	-1	1	0	1	1	1	0	0	0	0	0	0	0	1	1	0	0	0	0
		Swiss	6	6	0	4	2	2	1	1	0	5	5	0	3	3	0	1	1	0	0	0	0	1	1	0
Chêne-Bougeries	Female	Non Swiss	8	8	0	9	10	-1	9	9	0	6	6	0	20	21	-1	12	9	3	12	11	1	13	11	2
		Swiss	82	77	5	69	57	12	82	77	5	66	60	6	73	69	4	68	60	8	95	78	17	77	74	3
	Male	Non Swiss	9	9	0	9	8	1	10	9	1	12	12	0	17	15	2	6	4	2	11	10	1	9	7	2
		Swiss	43	39	4	34	32	2	40	37	3	42	36	6	44	37	7	48	44	4	46	44	2	41	38	3
Chêne-Bourg	Female	Non Swiss	6	6	0	3	1	2	7	8	-1	3	3	0	6	4	2	5	4	1	4	5	-1	8	6	2
		Swiss	13	11	2	16	16	0	25	20	5	11	7	4	12	10	2	11	9	2	15	15	0	10	9	1
	Male	Non Swiss	6	4	2	9	8	1	10	7	3	8	8	0	6	7	-1	8	5	3	2	1	1	5	3	2
		Swiss	14	12	2	29	25	4	19	17	2	19	18	1	20	18	2	11	10	1	17	15	2	14	11	3
Collex-Bossy	Female	Non Swiss	0	0	0	0	0	0	0	0	0	0	0	0	0	0	0	0	0	0	0	0	0	0	0	0
		Swiss	4	4	0	2	2	0	1	1	0	2	2	0	1	1	0	2	1	1	1	1	0	0	0	0
	Male	Non Swiss	0	0	0	0	0	0	0	0	0	0	0	0	0	0	0	0	0	0	2	2	0	0	0	0
		Swiss	5	5	0	4	4	0	3	2	1	3	3	0	3	3	0	3	2	1	1	1	0	2	2	0
Collonge-Bellerive	Female	Non Swiss	3	2	1	4	4	0	1	1	0	6	5	1	0	0	0	3	3	0	1	1	0	3	2	1
		Swiss	25	24	1	17	15	2	16	14	2	12	11	1	17	16	1	20	19	1	23	20	3	16	14	2
	Male	Non Swiss	3	3	0	7	4	3	10	9	1	2	2	0	3	4	-1	5	4	1	3	3	0	4	2	2
		Swiss	20	19	1	17	13	4	17	14	3	13	10	3	16	13	3	18	13	5	17	16	1	13	11	2
Cologny	Female	Non Swiss	3	3	0	3	3	0	6	5	1	4	3	1	6	7	-1	6	5	1	3	2	1	5	4	1
		Swiss	33	28	5	21	19	2	29	27	2	19	16	3	25	22	3	23	22	1	34	32	2	15	13	2
	Male	Non Swiss	2	3	-1	4	5	-1	1	0	1	5	2	3	7	6	1	7	5	2	8	9	-1	5	4	1
		Swiss	14	14	0	12	12	0	17	16	1	20	18	2	20	20	0	17	16	1	13	11	2	10	8	2
Confignon	Female	Non Swiss	0	0	0	2	2	0	2	1	1	0	1	-1	3	3	0	3	3	0	7	6	1	0	0	0
		Swiss	11	10	1	6	5	1	4	4	0	15	15	0	10	10	0	11	11	0	10	9	1	12	12	0
	Male	Non Swiss	4	5	-1	1	1	0	2	1	1	2	2	0	0	0	0	2	2	0	5	4	1	1	0	1
		Swiss	6	5	1	9	10	-1	9	7	2	10	8	2	3	2	1	14	14	0	7	6	1	13	10	3
Corsier	Female	Non Swiss	1	1	0	0	0	0	0	0	0	0	0	0	0	0	0	0	0	0	0	0	0	0	0	0
		Swiss	2	2	0	4	3	1	2	2	0	2	2	0	1	1	0	1	1	0	4	3	1	4	3	1
	Male	Non Swiss	1	1	0	0	0	0	0	0	0	1	1	0	0	1	-1	0	0	0	1	0	1	0	0	0
		Swiss	3	2	1	3	2	1	3	3	0	2	1	1	2	2	0	4	4	0	1	1	0	6	5	1
Céligny	Female	Non Swiss	0	0	0	0	0	0	1	1	0	0	0	0	1	0	1	1	1	0	0	0	0	0	0	0
		Swiss	1	1	0	0	0	0	0	0	0	0	0	0	5	3	2	1	0	1	1	1	0	2	2	0
	Male	Non Swiss	0	0	0	0	0	0	0	0	0	1	1	0	0	0	0	0	0	0	0	0	0	1	0	1
		Swiss	2	1	1	1	1	0	1	1	0	1	1	0	2	0	2	1	1	0	1	1	0	1	1	0
Dardagny	Female	Non Swiss	0	0	0	0	0	0	0	0	0	0	0	0	0	0	0	0	0	0	0	0	0	1	0	1
		Swiss	4	5	-1	2	2	0	1	1	0	2	2	0	0	0	0	1	1	0	1	1	0	3	3	0
	Male	Non Swiss	2	2	0	0	0	0	0	0	0	1	1	0	1	0	1	2	1	1	0	0	0	1	0	1
		Swiss	1	1	0	4	3	1	1	1	0	2	2	0	6	4	2	3	3	0	4	3	1	4	3	1

Municipality	Gender	Natl.	2009 FSO	2009 S	2009 Δ	2010 FSO	2010 S	2010 Δ	2011 FSO	2011 S	2011 Δ	2012 FSO	2012 S	2012 Δ	2013 FSO	2013 S	2013 Δ	2014 FSO	2014 S	2014 Δ	2015 FSO	2015 S	2015 Δ	2016 FSO	2016 S	2016 Δ
Genthod	Female	Non Swiss	1	1	0	2	2	0	1	1	0	0	0	0	1	1	0	1	1	0	0	1	-1	1	0	1
		Swiss	2	2	0	6	6	0	4	4	0	8	5	3	6	7	-1	3	2	1	8	7	1	9	8	1
	Male	Non Swiss	4	3	1	3	2	1	3	3	0	0	0	0	2	2	0	4	3	1	1	0	1	1	1	0
		Swiss	6	5	1	9	5	4	5	5	0	6	5	1	3	3	0	4	4	0	5	5	0	7	7	0
Genève-Cité	Female	Non Swiss	128	124	4	118	104	14	134	117	17	143	129	14	149	143	6	138	119	19	157	138	19	129	113	16
		Swiss	577	12	565	610	33	577	607	40	567	639	57	582	644	53	591	586	39	547	639	65	574	602	38	564
	Male	Non Swiss	157	161	-4	181	154	27	147	143	4	159	144	15	176	161	15	181	159	22	177	158	19	199	176	23
		Swiss	493	174	319	457	140	317	461	145	316	412	76	336	480	166	314	454	149	305	460	143	317	468	164	304
Genève-Eaux-Vives	Female	Non Swiss	128	124	4	118	104	14	134	117	17	143	129	14	149	143	6	138	119	19	157	138	19	129	113	16
		Swiss	577	12	565	610	33	577	607	40	567	639	57	582	644	53	591	586	39	547	639	65	574	602	38	564
	Male	Non Swiss	157	161	-4	181	154	27	147	143	4	159	144	15	176	161	15	181	159	22	177	158	19	199	176	23
		Swiss	493	174	319	457	140	317	461	145	316	412	76	336	480	166	314	454	149	305	460	143	317	468	164	304
Genève-Petit-Saconnex	Female	Non Swiss	128	124	4	118	104	14	134	117	17	143	129	14	149	143	6	138	119	19	157	138	19	129	113	16
		Swiss	577	12	565	610	33	577	607	40	567	639	57	582	644	53	591	586	39	547	639	65	574	602	38	564
	Male	Non Swiss	157	161	-4	181	154	27	147	143	4	159	144	15	176	161	15	181	159	22	177	158	19	199	176	23
		Swiss	493	174	319	457	140	317	461	145	316	412	76	336	480	166	314	454	149	305	460	143	317	468	164	304
Genève-Plainpalais	Female	Non Swiss	128	124	4	118	104	14	134	117	17	143	129	14	149	143	6	138	119	19	157	138	19	129	113	16
		Swiss	577	12	565	610	33	577	607	40	567	639	57	582	644	53	591	586	39	547	639	65	574	602	38	564
	Male	Non Swiss	157	161	-4	181	154	27	147	143	4	159	144	15	176	161	15	181	159	22	177	158	19	199	176	23
		Swiss	493	174	319	457	140	317	461	145	316	412	76	336	480	166	314	454	149	305	460	143	317	468	164	304
Grand-Saconnex	Female	Non Swiss	3	3	0	6	6	0	9	9	0	17	15	2	5	6	-1	10	8	2	6	4	2	10	9	1
		Swiss	31	29	2	31	27	4	29	25	4	27	26	1	30	25	5	23	19	4	29	27	2	24	22	2
	Male	Non Swiss	6	6	0	11	14	-3	12	11	1	5	3	2	8	8	0	6	6	0	9	8	1	10	7	3
		Swiss	27	25	2	26	25	1	13	12	1	22	17	5	20	18	2	22	18	4	18	18	0	22	18	4
Gy	Female	Non Swiss	0	0	0	0	0	0	1	0	1	0	0	0	0	0	0	0	0	0	0	0	0	0	0	0
		Swiss	0	0	0	0	0	0	1	1	0	1	1	0	3	3	0	1	1	0	1	1	0	0	0	0
	Male	Non Swiss	0	0	0	0	0	0	1	1	0	0	0	0	0	0	0	0	0	0	0	0	0	0	0	0
		Swiss	4	4	0	0	0	0	1	1	0	2	2	0	1	1	0	0	0	0	1	1	0	0	0	0
Hermance	Female	Non Swiss	2	2	0	0	0	0	0	0	0	2	2	0	0	0	0	0	0	0	3	3	0	0	0	0
		Swiss	7	7	0	10	9	1	4	4	0	9	9	0	9	8	1	4	4	0	5	5	0	9	9	0
	Male	Non Swiss	1	1	0	0	0	0	0	0	0	1	1	0	0	0	0	0	0	0	0	0	0	0	0	0
		Swiss	2	2	0	4	3	1	3	3	0	7	5	2	4	3	1	4	4	0	5	5	0	4	2	2
Jussy	Female	Non Swiss	0	0	0	0	0	0	0	0	0	0	0	0	0	0	0	0	0	0	0	0	0	0	0	0
		Swiss	1	1	0	3	2	1	3	3	0	3	3	0	2	2	0	3	3	0	3	2	1	6	6	0
	Male	Non Swiss	1	1	0	1	1	0	2	2	0	1	1	0	0	0	0	1	1	0	1	0	1	0	0	0
		Swiss	6	5	1	2	2	0	1	0	1	2	2	0	4	4	0	3	3	0	1	1	0	2	2	0
Laconnex	Female	Non Swiss	0	0	0	0	0	0	0	0	0	0	0	0	0	0	0	0	0	0	0	0	0	0	0	0
		Swiss	1	1	0	1	0	1	3	3	0	0	0	0	0	0	0	0	0	0	0	0	0	2	2	0
	Male	Non Swiss	0	0	0	0	0	0	1	0	1	0	0	0	0	0	0	1	0	1	0	0	0	0	0	0
		Swiss	1	1	0	0	0	0	1	1	0	1	1	0	3	3	0	0	0	0	1	1	0	0	0	0
Lancy	Female	Non Swiss	9	7	2	13	12	1	7	8	-1	24	21	3	9	10	-1	20	13	7	13	9	4	21	18	3
		Swiss	74	69	5	68	66	2	72	67	5	74	65	9	65	57	8	92	85	7	72	66	6	72	66	6
	Male	Non Swiss	17	19	-2	15	15	0	22	18	4	16	12	4	25	23	2	25	22	3	28	22	6	15	14	1
		Swiss	63	57	6	79	69	10	65	59	6	67	61	6	66	62	4	81	71	10	75	66	9	80	70	10

Municipality	Gender	Natl.	2009 FSO	2009 S	2009 Δ	2010 FSO	2010 S	2010 Δ	2011 FSO	2011 S	2011 Δ	2012 FSO	2012 S	2012 Δ	2013 FSO	2013 S	2013 Δ	2014 FSO	2014 S	2014 Δ	2015 FSO	2015 S	2015 Δ	2016 FSO	2016 S	2016 Δ
Meinier	Female	Non Swiss	0	0	0	0	0	0	0	0	0	1	1	0	1	0	1	1	1	0	1	1	0	0	0	0
		Swiss	6	6	0	3	2	1	2	1	1	3	3	0	3	2	1	4	4	0	1	1	0	2	2	0
	Male	Non Swiss	0	0	0	0	0	0	0	0	0	1	1	0	0	0	0	1	1	0	1	1	0	1	1	0
		Swiss	4	4	0	1	1	0	7	7	0	3	3	0	1	1	0	4	4	0	6	5	1	5	5	0
Meyrin	Female	Non Swiss	8	9	-1	8	6	2	11	10	1	12	13	-1	14	15	-1	13	10	3	13	11	2	15	12	3
		Swiss	36	30	6	38	36	2	40	37	3	55	47	8	55	46	9	35	35	0	59	54	5	40	39	1
	Male	Non Swiss	16	16	0	17	14	3	18	16	2	21	19	2	25	19	6	13	12	1	18	16	2	20	19	1
		Swiss	48	43	5	39	36	3	45	40	5	49	40	9	48	39	9	36	32	4	51	44	7	27	24	3
Onex	Female	Non Swiss	9	9	0	4	4	0	10	8	2	5	6	-1	10	9	1	13	9	4	14	13	1	16	10	6
		Swiss	70	67	3	64	59	5	63	59	4	71	65	6	68	62	6	53	49	4	84	72	12	70	65	5
	Male	Non Swiss	26	24	2	11	10	1	12	12	0	12	9	3	16	15	1	19	17	2	24	22	2	20	18	2
		Swiss	43	40	3	57	52	5	41	39	2	62	51	11	52	46	6	53	48	5	54	50	4	58	49	9
Perly-Certoux	Female	Non Swiss	1	1	0	0	0	0	2	2	0	0	0	0	2	1	1	0	0	0	0	0	0	2	2	0
		Swiss	1	1	0	2	2	0	4	4	0	3	2	1	2	2	0	3	3	0	3	3	0	6	4	2
	Male	Non Swiss	2	2	0	1	0	1	1	1	0	1	1	0	2	1	1	1	1	0	3	3	0	2	2	0
		Swiss	7	6	1	3	2	1	4	4	0	5	6	-1	7	7	0	11	9	2	4	4	0	6	5	1
Plan-les-Ouates	Female	Non Swiss	3	3	0	6	6	0	3	1	2	1	1	0	6	2	4	6	6	0	1	1	0	5	4	1
		Swiss	19	18	1	20	17	3	16	16	0	17	15	2	23	25	-2	23	19	4	27	21	6	15	14	1
	Male	Non Swiss	3	4	-1	5	3	2	2	2	0	10	8	2	5	4	1	8	8	0	3	2	1	2	2	0
		Swiss	12	8	4	29	24	5	12	10	2	22	18	4	19	19	0	17	15	2	23	21	2	24	22	2
Pregny-Chambésy	Female	Non Swiss	2	2	0	5	4	1	3	2	1	0	0	0	2	1	1	0	1	-1	3	2	1	2	1	1
		Swiss	17	17	0	17	12	5	13	13	0	10	8	2	17	17	0	19	14	5	18	16	2	18	17	1
	Male	Non Swiss	2	3	-1	2	2	0	1	2	-1	2	1	1	3	1	2	3	2	1	4	4	0	1	1	0
		Swiss	18	17	1	10	9	1	5	4	1	8	7	1	13	12	1	9	8	1	12	10	2	11	11	0
Presinge	Female	Non Swiss	1	1	0	2	2	0	2	2	0	3	3	0	2	2	0	1	1	0	2	2	0	1	1	0
		Swiss	11	11	0	7	7	0	6	4	2	3	3	0	10	10	0	9	8	1	3	3	0	3	3	0
	Male	Non Swiss	1	0	1	2	2	0	1	1	0	1	1	0	1	1	0	3	3	0	1	1	0	2	1	1
		Swiss	3	2	1	5	5	0	5	4	1	6	6	0	9	8	1	6	6	0	7	6	1	8	7	1
Puplinge	Female	Non Swiss	1	0	1	0	0	0	2	2	0	0	0	0	0	0	0	1	1	0	0	0	0	0	0	0
		Swiss	6	6	0	3	2	1	3	2	1	2	2	0	6	6	0	4	4	0	3	3	0	5	5	0
	Male	Non Swiss	0	0	0	3	3	0	2	1	1	0	0	0	2	1	1	0	0	0	1	1	0	2	2	0
		Swiss	6	6	0	0	0	0	3	2	1	8	5	3	4	5	-1	6	6	0	4	4	0	4	2	2
Russin	Female	Non Swiss	0	0	0	0	0	0	0	0	0	0	0	0	0	0	0	0	0	0	0	0	0	1	1	0
		Swiss	1	0	1	1	1	0	1	1	0	0	0	0	1	1	0	0	0	0	1	1	0	0	0	0
	Male	Non Swiss	0	0	0	0	0	0	0	0	0	0	0	0	0	0	0	0	0	0	0	0	0	0	0	0
		Swiss	0	0	0	1	1	0	0	0	0	3	3	0	1	1	0	0	0	0	2	1	1	0	0	0
Satigny	Female	Non Swiss	2	2	0	0	0	0	0	0	0	3	3	0	5	4	1	0	0	0	0	0	0	1	1	0
		Swiss	8	7	1	11	10	1	15	15	0	13	8	5	15	16	-1	12	8	4	20	18	2	16	15	1
	Male	Non Swiss	2	2	0	3	3	0	4	2	2	3	2	1	4	4	0	3	4	-1	3	2	1	1	1	0
		Swiss	16	13	3	10	9	1	5	6	-1	10	9	1	14	13	1	11	9	2	8	7	1	9	10	-1
Soral	Female	Non Swiss	1	1	0	2	2	0	0	0	0	0	0	0	2	2	0	0	0	0	1	1	0	0	0	0
		Swiss	17	15	2	7	5	2	5	4	1	15	11	4	11	10	1	9	9	0	5	5	0	10	10	0
	Male	Non Swiss	0	0	0	0	0	0	0	0	0	1	0	1	0	0	0	1	1	0	1	1	0	2	2	0
		Swiss	6	5	1	7	3	4	4	4	0	2	2	0	7	7	0	3	2	1	7	7	0	4	4	0

			2009			2010			2011			2012			2013			2014			2015			2016		
Municipality	Gender	Natl.	FSO	S	Δ	FSO	S	Δ	FSO	S	Δ	FSO	S	Δ	FSO	S	Δ	FSO	S	Δ	FSO	S	Δ	FSO	S	Δ
Thônex	Female	Non Swiss	11	12	-1	7	4	3	8	5	3	9	7	2	6	8	-2	5	4	1	10	8	2	6	4	2
		Swiss	35	34	1	34	34	0	27	24	3	32	30	2	43	36	7	27	25	2	41	38	3	31	30	1
	Male	Non Swiss	9	10	-1	13	11	2	4	4	0	10	11	-1	19	13	6	9	9	0	15	15	0	8	8	0
		Swiss	25	20	5	31	28	3	36	34	2	32	29	3	33	27	6	23	19	4	36	30	6	36	31	5
Troinex	Female	Non Swiss	0	0	0	1	1	0	1	1	0	0	0	0	0	0	0	0	0	0	0	0	0	0	0	0
		Swiss	5	5	0	1	1	0	4	4	0	3	3	0	1	0	1	4	4	0	5	5	0	7	5	2
	Male	Non Swiss	1	0	1	0	0	0	0	0	0	2	2	0	0	0	0	1	1	0	0	0	0	0	0	0
		Swiss	2	3	-1	3	2	1	4	3	1	7	5	2	7	7	0	3	2	1	1	1	0	6	3	3
Vandoeuvres	Female	Non Swiss	0	0	0	1	1	0	2	2	0	1	1	0	1	1	0	1	1	0	1	0	1	1	1	0
		Swiss	12	12	0	9	7	2	6	6	0	5	6	-1	18	16	2	8	8	0	5	5	0	9	9	0
	Male	Non Swiss	1	1	0	2	2	0	2	2	0	0	0	0	2	2	0	2	1	1	0	0	0	2	1	1
		Swiss	7	7	0	8	7	1	7	5	2	7	5	2	7	7	0	7	7	0	9	7	2	7	7	0
Vernier	Female	Non Swiss	17	18	-1	18	11	7	21	20	1	22	19	3	17	18	-1	14	11	3	30	22	8	19	14	5
		Swiss	60	49	11	56	46	10	60	56	4	50	44	6	61	59	2	64	59	5	87	78	9	59	53	6
	Male	Non Swiss	29	26	3	18	16	2	31	28	3	34	27	7	29	29	0	37	29	8	39	36	3	32	33	-1
		Swiss	66	58	8	86	79	7	82	73	9	73	61	12	69	61	8	60	47	13	71	55	16	83	72	11
Versoix	Female	Non Swiss	6	6	0	5	5	0	10	7	3	5	7	-2	9	5	4	12	10	2	8	7	1	5	4	1
		Swiss	34	31	3	24	22	2	30	30	0	42	37	5	45	37	8	49	44	5	47	38	9	32	28	4
	Male	Non Swiss	7	6	1	8	6	2	15	12	3	4	2	2	7	5	2	11	9	2	6	3	3	11	11	0
		Swiss	23	21	2	28	24	4	32	26	6	33	28	5	35	31	4	37	31	6	32	29	3	33	23	10
Veyrier	Female	Non Swiss	10	8	2	8	9	-1	4	4	0	9	8	1	4	2	2	5	3	2	9	9	0	13	12	1
		Swiss	55	54	1	81	69	12	66	64	2	64	59	5	86	78	8	69	62	7	86	75	11	73	69	4
	Male	Non Swiss	4	5	-1	8	5	3	5	4	1	10	6	4	11	8	3	11	8	3	5	3	2	11	10	1
		Swiss	44	38	6	46	36	10	35	30	5	53	47	6	57	54	3	45	42	3	51	45	6	42	37	5

Figure S4: Difference in Years of Potential Life Lost or Gained (YPLLG) distribution when using the cohort Life Expectancy at Birth (LEB) (red) or the period LEB (blue) as the reference value, where the YPLLG is the difference between the age at death and the individual cohort LEB. Although we did not compare the geographic footprint of YPLLG obtained using the period LEB rather than the cohort LEB, the graph suggests that this would not have significantly affected the results.

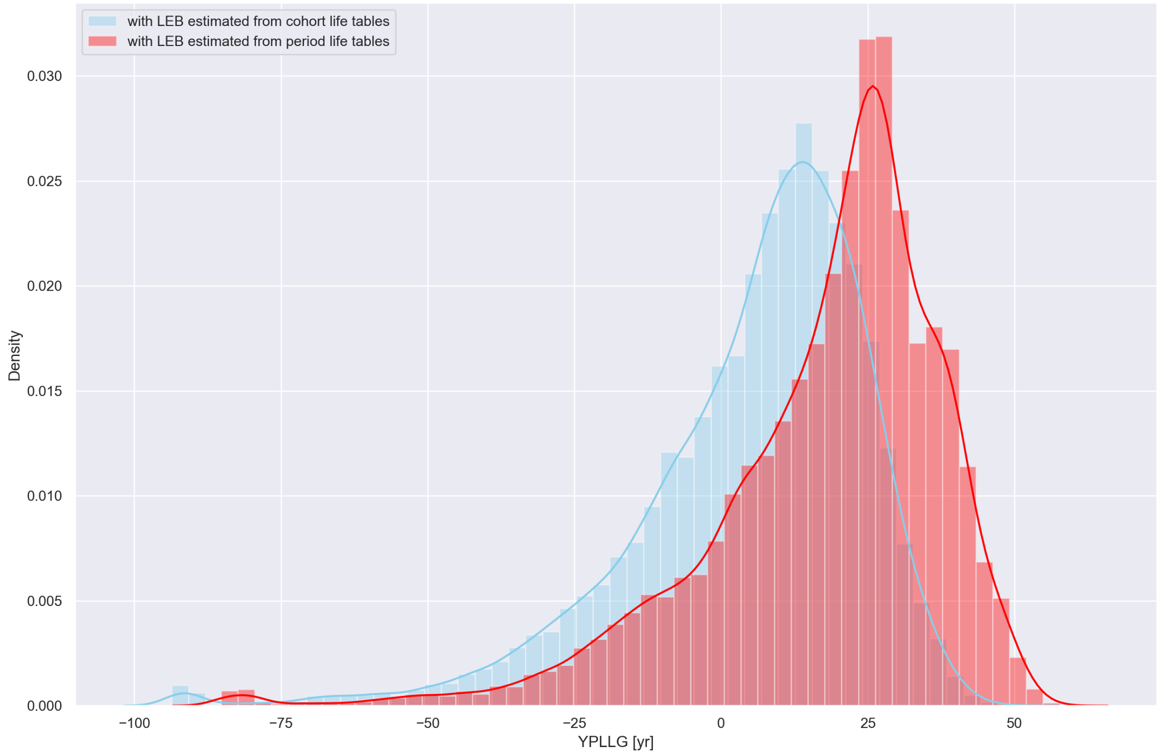

References

[1] Roger Koenker. *Quantreg: Quantile Regression*, 2021.

[2] Katharine M McGreevy, Stuart R Lipsitz, A Linder, Eric Rimm, and David G Hoel. Using Median Regression to Obtain Adjusted Estimates of Central Tendency for Skewed Laboratory and Epidemiologic Data. page 6, 2008.
